# Role of Pex31 in metabolic adaptation of the nucleus–vacuole junction

**DOI:** 10.1242/jcs.264171

**Published:** 2025-11-21

**Authors:** Marie Hugenroth, Pascal Höhne, Xue-Tong Zhao, Mike Wälte, Duy Trong Vien Diep, Rebecca Martina Fausten, Maria Bohnert

**Affiliations:** ^1^ Institute of Cell Dynamics and Imaging, University of Münster, Von-Esmarch-Str. 56, 48149 Münster, Germany; ^2^ Cells in Motion Interfaculty Centre (CiM), University of Münster, 48149 Münster, Germany

**Keywords:** Pex31, Pex30, Nsg1, Nsg2, Tcb1, Shr5

## Abstract

The nucleus–vacuole junction (NVJ) in *Saccharomyces cerevisiae* is a multifunctional contact site between the membrane of the nuclear endoplasmic reticulum (ER) and the vacuole that has diverse roles in lipid metabolism, transfer and storage. Adaptation of NVJ functions to metabolic cues is mediated by a striking remodeling of the size and the proteome of the contact site, but the extent and the molecular determinants of this plasticity are not fully understood. Using microscopy-based screens, we monitored NVJ remodeling in response to glucose availability. We identified Pex31, Nsg1, Nsg2, Shr5, and Tcb1 as NVJ residents. Glucose starvation typically results in an expansion of the NVJ size and proteome. Pex31 shows an atypical behavior, being specifically enriched at the NVJ in high-glucose conditions. Loss of Pex31 uncouples NVJ remodeling from glucose availability, resulting in recruitment of glucose starvation-specific residents and NVJ expansion at glucose-replete conditions. Moreover, *PEX31* deletion results in alterations of sterol ester storage and a remodeling of vacuolar membranes that phenocopy glucose starvation responses. We conclude that Pex31 has a role in metabolic adaptation of the NVJ.

## Teaser

**Summary:** Using microscopy-based screens in yeast, we identified Pex31, Nsg1, Nsg2, Shr5 and Tcb1 as residents of the nucleus–vacuole junction (NVJ). Pex31 has a role in NVJ adaptation to glucose availability.

## INTRODUCTION

Eukaryotic cells are equipped with a set of membrane-bounded organelles that provide distinct, biochemically optimized compartments. Organelle functions are coordinated via contact sites, structures at which different organelles are physically linked by tether machineries to mediate selective material transfer, to enable signal transmission and to locally organize enzymatic activities (Eisenberg-Bord et al., 2016; Scorrano et al., 2019). The cellular contact site landscape is not static. Instead, metabolic alterations and environmental cues promote reorganizations of the extent, composition and functions of contact sites to enable coordinated cellular adaptations (Bohnert, 2020).

A prominent contact site in *Saccharomyces cerevisiae* (from here on referred to as yeast) is the nucleus–vacuole junction (NVJ). This structure links the nuclear endoplasmic reticulum (ER) and the vacuole (yeast lysosome) via an interaction of the nuclear ER protein Nvj1 and the vacuole surface protein Vac8 (Pan et al., 2000). The NVJ is a multifunctional contact site. Originally described as a structure involved in a special form of autophagy termed piecemeal microautophagy of the nucleus (PMN; Roberts et al., 2003), the NVJ has emerged as a central platform for lipid handling. Several NVJ-resident proteins involved in diverse branches of lipid metabolism have been identified, including Tsc13, an enzyme involved in fatty acid elongation (Kohlwein et al., 2001); the fatty acyl-CoA synthetase Faa1 (Hariri et al., 2018); the 3-hydroxy-3-methylglutaryl-coenzyme A (HMG-CoA) reductases Hmg1 and Hmg2 (Rogers et al., 2021); the phosphatidic acid phosphatase Pah1 (Barbosa et al., 2015); and Cvm1 (Bisinski et al., 2022), a protein involved in sphingolipid metabolism. A further group of NVJ proteins is involved in inter-organelle lipid transfer: Osh1 (Elbaz-Alon et al., 2015; Gatta et al., 2015; Kvam and Goldfarb, 2004; Levine and Munro, 2001), Lam6 (Elbaz-Alon et al., 2015; Gatta et al., 2015; Murley et al., 2015), Nvj2 (Liu et al., 2017; Toulmay and Prinz, 2012) and Vps13 (Lang et al., 2015). Mdm1 is an NVJ component that has a role in the localized formation of lipid droplets, ER-derived storage organelles for neutral lipids (Hariri et al., 2018; Henne et al., 2015). Nvj3, a protein with structural similarities to Mdm1, is also an NVJ resident (Hariri et al., 2018; Henne et al., 2015). Two further NVJ proteins related to organelle biogenesis are Pex29 and Pex30 (Ferreira and Carvalho, 2021). These two proteins are part of a structurally related family of ER membrane proteins that also comprises three additional members, Pex28, Pex31 and Pex32. Originally, all family members were described as proteins required for regular abundance and morphology of peroxisomes (Vizeacoumar et al., 2003, 2004). Later, roles of Pex30 in biogenesis of pre-peroxisomal vesicles (Joshi et al., 2016) and lipid droplets (Choudhary et al., 2020; Joshi et al., 2018; Wang et al., 2018) from the ER membrane were described. In summary, the NVJ houses a set of proteins that fulfill multiple functions in lipid metabolism and transport, and in organelle biogenesis from the ER.

A prominent feature of the NVJ is its remarkable degree of metabolic adaptability. Although the NVJ is typically a small structure in nutrient-replete conditions, it strongly expands in response to different types of nutrient deprivation (Hariri et al., 2018; Roberts et al., 2003; Tosal-Castano et al., 2021). During glucose deprivation, this adaptation of the NVJ size depends on the NVJ-resident protein Snd3 (Tosal-Castano et al., 2021). Importantly, it is not only the size of the NVJ that adapts to metabolic cues, but also its proteome. Whereas some NVJ proteins, such as the main tether pair Nvj1–Vac8, are permanently present at the NVJ, a large fraction of the known NVJ proteins are conditional NVJ residents that are largely absent from the NVJ when nutrients are replete but accumulate at the contact site when cells run out of glucose. Such a glucose deprivation-induced NVJ recruitment has been described for Snd3 (Tosal-Castano et al., 2021), Hmg1 and Hmg2 (Rogers et al., 2021), Pah1 (Barbosa et al., 2015), Nvj2 (Toulmay and Prinz, 2012), Cvm1 (Bisinski et al., 2022), Faa1 (Hariri et al., 2018), Vps13 (Lang et al., 2015), and Pex29 and Pex30 (Ferreira and Carvalho, 2021). However, remodeling of the NVJ proteome has not been systematically studied, and the molecular determinants of the proteomic adaptation of the NVJ have been largely unclear.

Here, we used systematic microscopy-based approaches to compare the NVJ at glucose-replete and -restricted conditions and identified five additional NVJ proteins: the permanent NVJ resident Shr5 and the conditional residents Nsg1, Nsg2, Tcb1 and Pex31. Whereas Nsg1, Nsg2 and Tcb1 belong to the large group of conditional NVJ residents that accumulate at low-glucose conditions, Pex31 is unique as it is enriched at the NVJ at high-glucose conditions but not during glucose starvation. *PEX31* deletion results in a dysregulation of NVJ remodeling: low-glucose-specific NVJ features, such as NVJ enlargement and expansion of the NVJ proteome, occur independently of glucose availability in Δ*pex31* cells. We conclude that Pex31 has a role in NVJ adaptation to metabolic cues.

## RESULTS

### A microscopy-based screen identifies residents of the NVJ

**Fig. 1.**
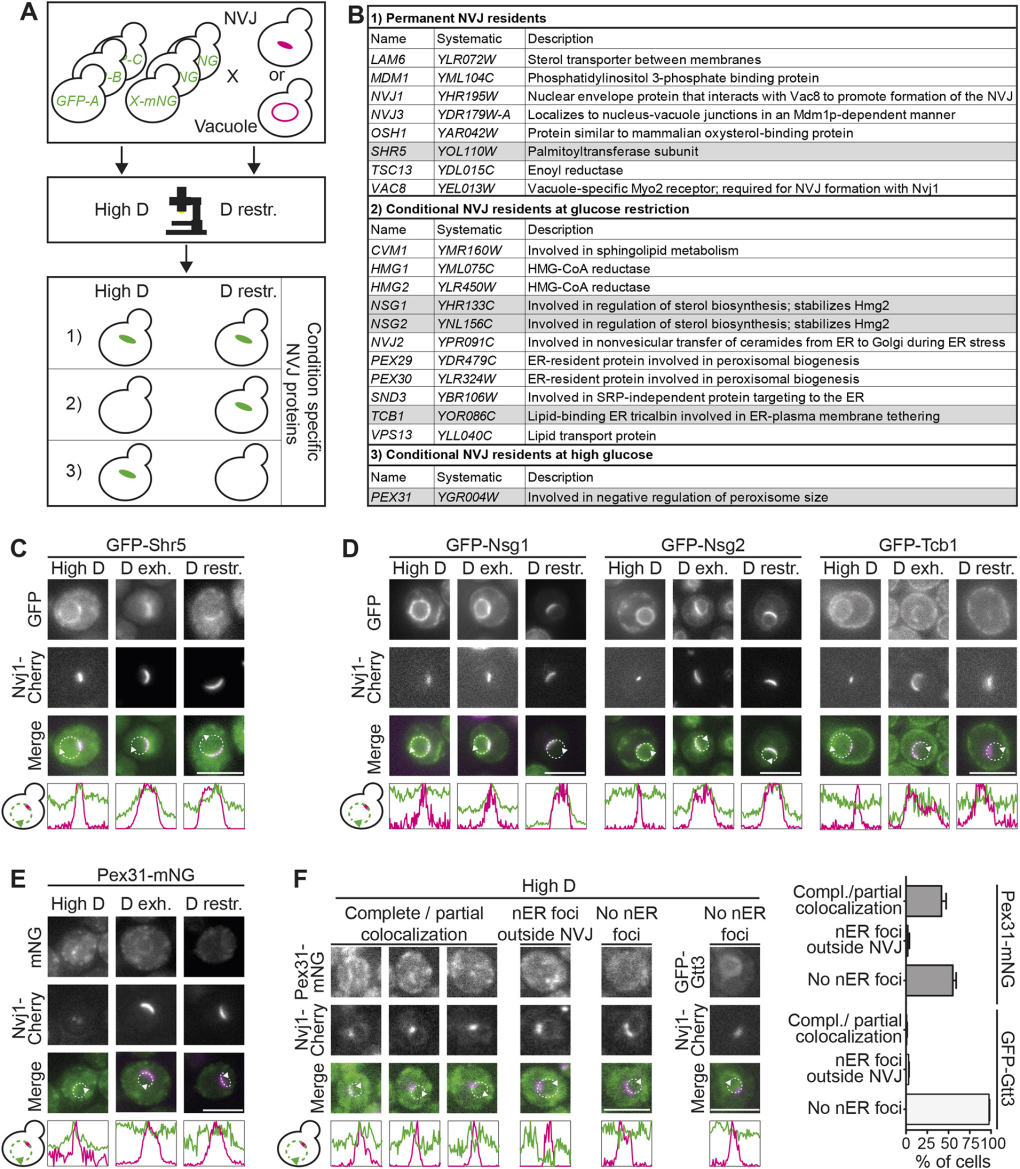
**A microscopy-based screen identifies Pex31, Nsg1, Nsg2, Shr5 and Tcb1 as residents of the NVJ.** (A) Schematic depiction of a screen searching for NVJ residents in glucose-replete and glucose-restricted conditions. Nvj1–Cherry or Zrc1–Cherry were introduced as markers of the NVJ into a collection of strains expressing N-terminally GFP-tagged or C-terminally mNG-tagged proteins related to lipid handling. Strains were imaged in glucose-replete (High D) and glucose-restricted (D restr.) conditions and categorized according to their localization to the NVJ: (1) permanent NVJ residents displaying NVJ localization in both conditions, (2) conditional NVJ residents localizing to the NVJ upon glucose restriction and (3) conditional NVJ residents localizing to the NVJ in glucose-replete conditions. (B) List of hits from the screen in A. Previously described NVJ residents in white, additional NVJ residents in gray. (C) A strain expressing GFP–Shr5 and Nvj1–Cherry was grown in glucose-replete (High D), glucose-exhausted (D exh.) and glucose-restricted (D restr.) conditions. Brightness and contrast were adjusted individually. (D) Cells co-expressing GFP–Nsg1, GFP–Nsg2 or GFP–Tcb1 with Nvj1–Cherry were grown in glucose-replete (High D), glucose-exhausted (D exh.) and glucose-restricted (D restr.) conditions. Brightness and contrast were adjusted individually. (E) Pex31–mNG Nvj1–Cherry cells were grown in glucose-replete (High D), glucose-exhausted (D exh.) and glucose-restricted (D restr.) conditions. Brightness and contrast were adjusted individually. (F) Pex31–mNG Nvj1–Cherry and GFP–Gtt3 Nvj1–Cherry cells grown in glucose-replete conditions were categorized based on Pex31–mNG or GFP–Gtt3 localization in reference to the Nvj1–Cherry signal. Left: example images of each category are shown (nER, nuclear ER). Right: quantification of phenotypical categories depicted as mean±s.e.m. *N*=50 cells; *n*=3. In C–F, merge images and line graphs show GFP and mNG signals in green and Nvj1–Cherry in magenta. Dashed lines mark the nuclear ER and were used for the quantification of fluorescence intensity shown in the line graphs. Arrowheads mark the positions of the ends of the line graphs. Scale bars: 5 µm. Images in C–E are representative of at least three independent experiments.

Alterations in glucose availability result in pronounced adaptations of the NVJ proteome (Barbosa et al., 2015; Bisinski et al., 2022; Ferreira and Carvalho, 2021; Hariri et al., 2018; Lang et al., 2015; Rogers et al., 2021; Tosal-Castano et al., 2021; Toulmay and Prinz, 2012), but the extent of these remodeling processes and the underlying molecular determinants are only partially understood. To gain a broad view on NVJ remodeling, we performed a microscopy-based screen. We assembled a mutant collection of 337 strains (Table S1) expressing N-terminally GFP-tagged proteins from a genome-wide mutant library (Weill et al., 2018; Yofe et al., 2016). The collection comprised known NVJ residents and their paralogs as well as proteins involved in lipid metabolism and lipid transport. We then used an automated mating approach (Cohen and Schuldiner, 2011; Tong and Boone, 2006) to introduce the NVJ marker Nvj1–mCherry (hereafter Nvj1–Cherry) into this mutant collection. In a parallel approach, we used the vacuolar membrane marker Zrc1–mCherry (hereafter Zrc1–Cherry) as an alternative way to visualize the vacuole–nuclear ER interface without manipulation of an integral NVJ protein. We complemented these mutant collections with select manually created strains expressing C-terminally mNeonGreen (mNG)-tagged proteins (Fig. 1A; Table S1). We also included a strain expressing a Vps13 variant internally tagged with GFP that was previously described as a functional NVJ protein (Lang et al., 2015). All strains were analyzed by automated microscopy in two different metabolic conditions: cells were either grown overnight on glucose-replete medium, back-diluted to fresh glucose-replete medium and further grown to logarithmic growth phase (Fig. 1A, High D), or precultured on glucose-replete medium overnight and then incubated for 4 h in glucose restriction medium containing only 0.001% glucose (Fig. 1A, D restr.). GFP- or mNG-tagged proteins were then classified according to their localization at the NVJ (Fig. 1B; Fig. S1A–D). Overall, we identified 20 NVJ residents, five of which have, to our knowledge, not been described before. Eight proteins were present at the NVJ at both high- and low-glucose conditions (Fig. 1B, permanent NVJ residents), 11 accumulated at the NVJ specifically upon glucose restriction (Fig. 1B, conditional NVJ resident at glucose restriction) and one was found enriched at the NVJ selectively in the glucose-replete condition (Fig. 1B, conditional NVJ residents at high glucose). The five previously unknown NVJ proteins (Fig. 1B, gray boxes) were re-analyzed in the high-glucose condition and in the acute glucose restriction condition used in the screen. Additionally, we included a protocol for a gradual form of glucose starvation, by growing cells in medium supplemented with 2% glucose for 20–22 h into early stationary phase, resulting in glucose exhaustion from the medium (D exh.). Shr5 has previously been described as an ER-resident protein involved in palmitoylation and correct targeting of Ras2 (Dong et al., 2003; Jung et al., 1995; Lobo et al., 2002). We detected this protein at the NVJ at all three conditions analyzed (Fig. 1C; Fig. S1C), classifying it as a permanent NVJ resident (Fig. 1B), similar to the known NVJ proteins Nvj1, Nvj3, Mdm1, Osh1, Lam6, Tsc13 and Vac8 (Fig. S1A). Nsg1 and Nsg2 are two homologs of the mammalian insulin-induced gene (INSIG) proteins, which are involved in regulation of sterol levels (Flury et al., 2005). Nsg1 and Nsg2 have been found to bind to the sterol-sensing domain of the HMG-CoA reductase Hmg2, thereby stabilizing this enzyme that mediates the rate-limiting enzymatic step in the mevalonate pathway (Basson et al., 1986; Flury et al., 2005; Rogers et al., 2021). Both Hmg2 and its homolog Hmg1 have in the past been shown to localize to the NVJ specifically during glucose starvation (Rogers et al., 2021). We found that Nsg1 and Nsg2 show a similar behavior by accumulating at the NVJ specifically upon glucose exhaustion or restriction (Fig. 1D; Fig. S1D). A further protein that we found enriched at the NVJ in response to the low-glucose conditions, but not in the high-glucose condition, is Tcb1 (Fig. 1D; Fig. S1D). Tcb1 is part of the family of tricalbin proteins, which have been identified as residents of the contact site between the ER and the plasma membrane (Collado et al., 2019; Creutz et al., 2004; Manford et al., 2012; Toulmay and Prinz, 2012). In summary, we identify Tcb1, Nsg1 and Nsg2 as low-glucose NVJ proteins (Fig. 1B), similar to the previously described conditional NVJ residents Hmg1, Hmg2, Snd3, Nvj2, Cvm1, Vps13, Pex29 and Pex30 (Fig. S1B). The latter two proteins are part of a structurally related protein family (Vizeacoumar et al., 2003, 2004). Interestingly, we identified a further member of this family, Pex31, at the NVJ; however, this protein showed an inverse behavior, being enriched at the NVJ in glucose-replete conditions, but not in conditions of glucose restriction or exhaustion (Fig. 1E). We analyzed the behavior of this high-glucose-specific NVJ resident in more detail and detected a colocalization of Pex31 foci with Nvj1–Cherry in over 40% of cells at glucose repletion, whereas a control nuclear ER protein, Gtt3, showed virtually no NVJ-associated foci (Fig. 1F; Fig. S2A). Pex31 and its family members all have a characteristic domain structure, comprising an N-terminal reticulon homology domain (RHD) (Joshi et al., 2016) and a C-terminal dysferlin domain (Ferreira et al., 2025; House et al., 2025). We asked which of these Pex31 domains are required for localization at the NVJ and created Pex31 variants lacking either domain. Loss of the dysferlin domain resulted in a dispersed Pex31 distribution throughout the ER. A Pex31 variant lacking the membrane integral RHD showed a cytosolic localization, and fusion of this Pex31 variant with either the ER anchor of Ubc6 or an Scs2/Scs22-binding FFAT motif restored ER localization but not NVJ localization of the protein (Fig. S2B). Finally, we created a strain expressing Pex31–HA from its genomic locus under control of its own promoter and analyzed Pex31 protein levels. We found that Pex31 abundance dropped steeply upon glucose restriction as well as in glucose exhaustion conditions (Fig. S2C).

In summary, we have identified Shr5, Nsg1, Nsg2, Tcb1 and Pex31 as NVJ residents, of which Pex31 is uniquely enriched at the NVJ in the high-glucose condition.

### Pex31 limits the NVJ proteome in high-glucose conditions

**Fig. 2.**
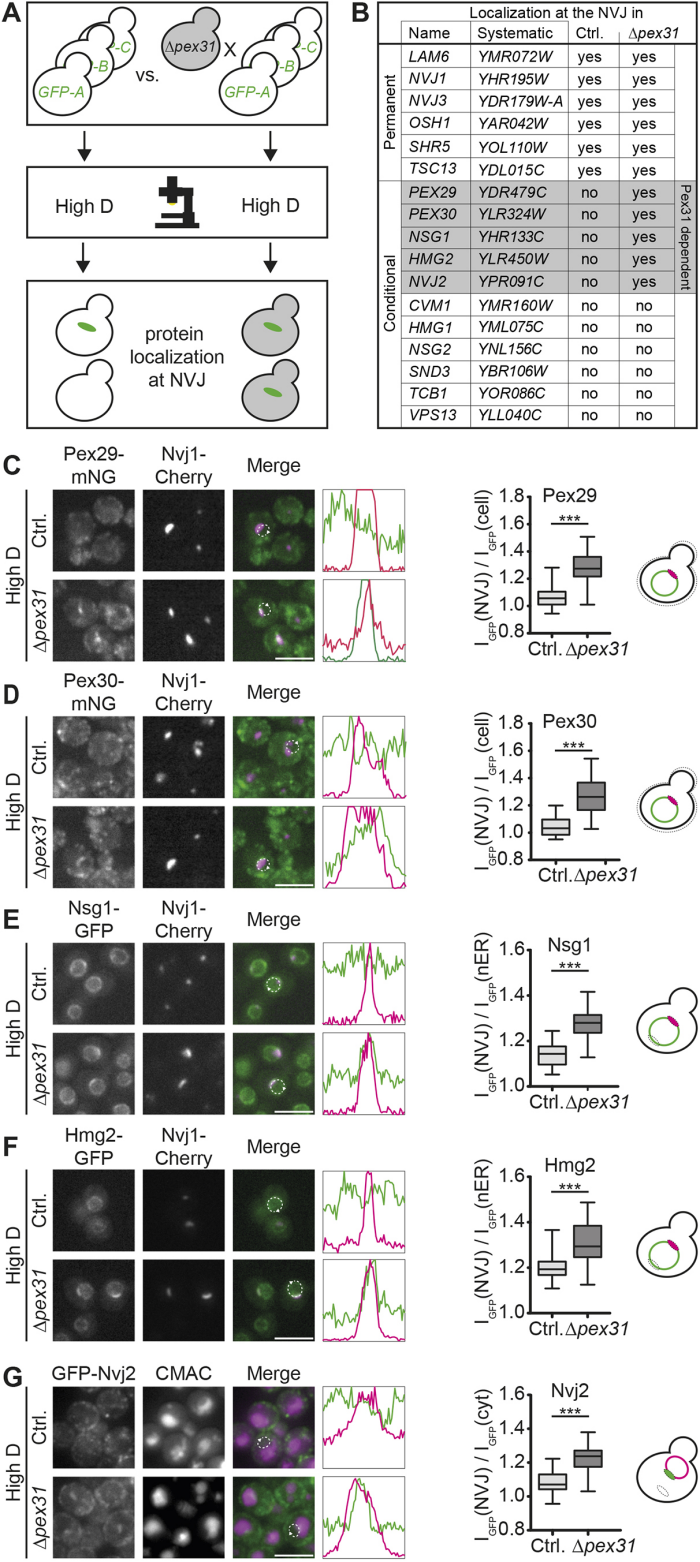
**Loss of Pex31 alters NVJ composition in glucose-replete conditions.** (A) Schematic representation of a screen comparing protein localization in the presence and absence of *PEX31*. Automated mating was used to delete *PEX31* in a genome-wide collection of strains expressing N-terminally GFP-tagged proteins under control of a *NOP1* promotor. The resulting mutant collection was imaged alongside the parent collection in glucose-replete conditions, and all strains were assessed for GFP signal at the NVJ. (B) List of hits from the screen. Marked in gray, proteins re-localizing to the NVJ in glucose-rich conditions following deletion of *PEX31*. Ctrl, control. (C) Left: Pex29–mNG Nvj1–Cherry (Ctrl.) and Pex29–mNG Δ*pex31* Nvj1–Cherry cells grown in glucose-replete conditions, with quantification of fluorescence intensity shown in the line graphs. Right: enrichment of mNG signal at the NVJ [I_GFP_(NVJ)] over whole-cell mNG signal [I_GFP_(cell)] was quantified. (D) Left: Pex30–mNG Nvj1–Cherry (Ctrl.) and Pex30–mNG Δ*pex31* Nvj1–Cherry cells grown in glucose-replete conditions, with quantification of fluorescence intensity shown in the line graphs. Right: quantification as in C. (E) Left: Nsg1–GFP Nvj1–Cherry (Ctrl.) and Nsg1–GFP Δ*pex31* Nvj1–Cherry cells grown in glucose-replete conditions, with quantification of fluorescence intensity shown in the line graphs. Right: enrichment of GFP signal at the NVJ [I_GFP_(NVJ)] over nuclear ER GFP signal at the opposite side [I_GFP_(nER)] was quantified. (F) Left: Hmg2–GFP and Nvj1–Cherry co-expressed in control (Ctrl.) and Δ*pex31* cells grown in glucose-replete conditions, with quantification of fluorescence intensity shown in the line graphs. Right: quantification as in E. (G) Left: GFP–Nvj2 (Ctrl.) and GFP–Nvj2 Δ*pex31* cells were stained with the vacuole lumen dye CMAC and imaged in glucose-replete conditions, with quantification of fluorescence intensity shown in the line graphs. Right: enrichment of GFP signal at the NVJ [I_GFP_(NVJ)] over cytosolic GFP signal [I_GFP_(cyt)] was quantified. In C–G, merge images and line graphs show GFP and mNG signals in green and Nvj1–Cherry in magenta. Dashed lines mark the nuclear ER and were used for the quantification of fluorescence intensity shown in the line graphs. Arrowheads mark the positions of the ends of the line graphs. Data represented in box plots show the median (line), interquartile range (box) and minimum to maximum values (whiskers). *N*=30 cells, *n*=3. Compared by two-tailed unpaired *t*-test. ***P<0.001. Scale bars: 5 µm.

The identification of Pex31 as an atypical NVJ resident with a preference for high-glucose conditions prompted us to follow up on this protein. To assess the impact of Pex31 on the NVJ, we introduced a Δ*pex31* allele into a mutant collection expressing N-terminally GFP-tagged protein variants (Weill et al., 2018; Yofe et al., 2016) by automated mating (Cohen and Schuldiner, 2011; Tong and Boone, 2006). As in the initial screen (Fig. 1A), the collection was complemented with manually created strains expressing selected C-terminally mNG-tagged proteins. We then cultured both the new mutant collection carrying the Δ*pex31* allele and the unaltered parent library on high-glucose medium to logarithmic growth phase, the condition where typically only the permanent NVJ proteins accumulate at the contact site. We analyzed both mutant collections by automated microscopy and compared localization of the tagged proteins (Fig. 2A). We found that localization of the permanent NVJ residents was unaffected by *PEX31* deletion (Fig. 2B, top). In contrast, we observed an effect of *PEX31* deletion on part of the conditional NVJ proteome. Pex29, Pex30, Nsg1, Hmg2 and Nvj2, which typically accumulate at the NVJ selectively in low-glucose conditions (Fig. 1B,D), showed an enrichment at the NVJ on high-glucose medium in response to the *PEX31* deletion (Fig. 2B, labeled in gray). We re-created all strains manually to re-assess and quantify localization of the five proteins. Proteins of interest were marked with a green fluorescent marker, and either the vacuole was visualized by CMAC staining (labeling the vacuole lumen) or the NVJ was marked by genomic expression of Nvj1–Cherry (Fig. 2C–G, left). All five proteins were found to be enriched at the NVJ in Δ*pex31* cells (Fig. 2C–G, right), confirming the results of the screen.

In summary, Pex31 is a resident of high-glucose NVJs, which typically have a limited proteome, and *PEX31* deletion results in an expansion of the high-glucose NVJ proteome in a manner that partially phenocopies low-glucose conditions.

### *PEX31* deletion and overexpression uncouple NVJ adaptation from glucose availability

**Fig. 3.**
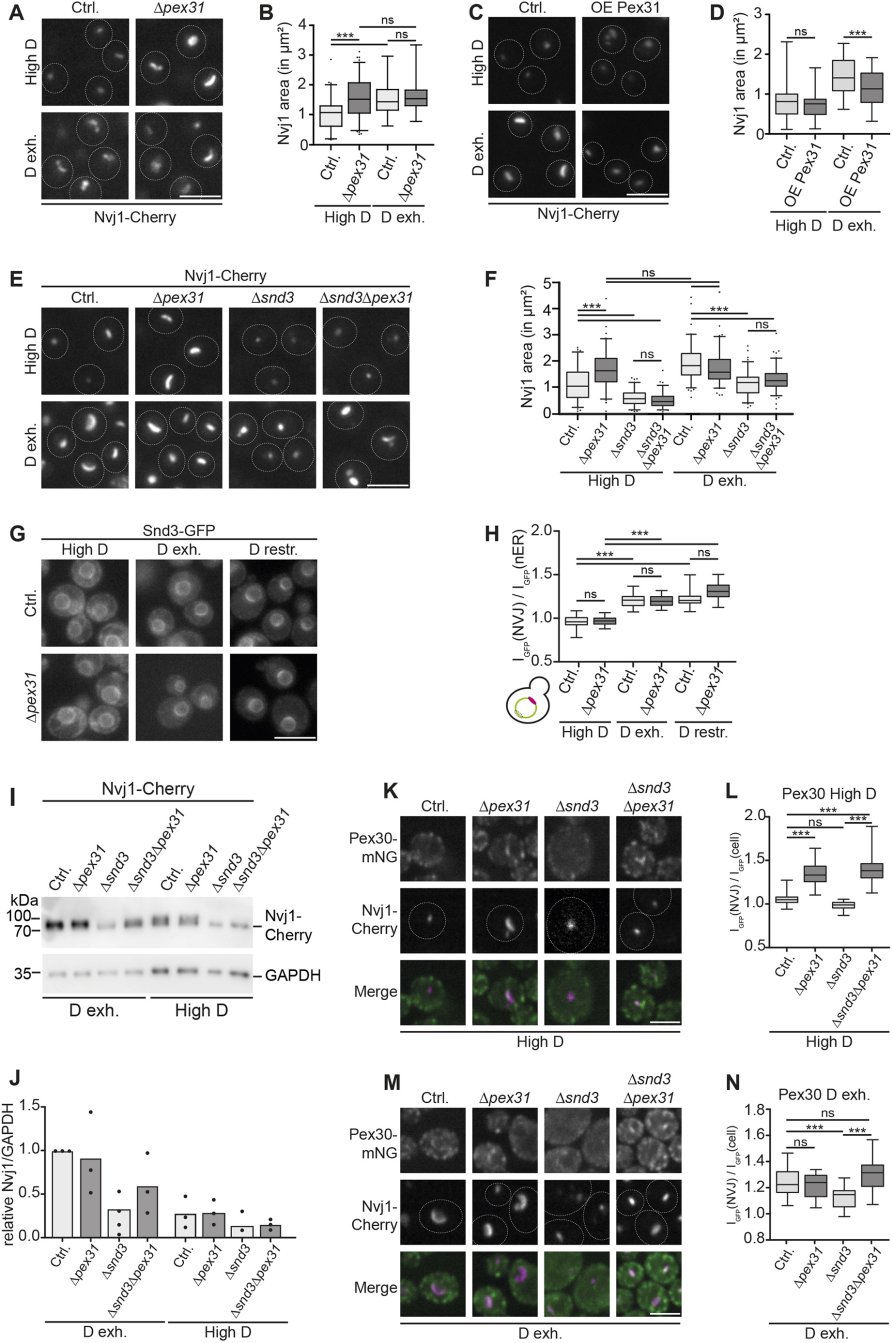
***PEX31* mutations uncouple NVJ adaptation from glucose availability.** (A) Control (Ctrl.) and Δ*pex31* cells expressing Nvj1–Cherry were grown in either glucose-replete (High D) or glucose-exhausted (D exh.) conditions. (B) Quantification of Nvj1–Cherry signal from A. Nvj1 area (in µm²) is depicted as boxplots showing the median (line), interquartile range (box) and 5–95 percentile range (whiskers). Compared by one-way ANOVA with Tukey's multiple comparisons. ns, not significant; ***P<0.001. *N*=50 cells, *n*=3. (C) Control and Pex31-overexpressing (OE Pex31) cells carrying an Nvj1–Cherry allele were cultured in glucose-replete or glucose-exhaustion conditions and analyzed by microscopy. (D) Quantification of cells from C. Nvj1 area (in µm²) is depicted as boxplots showing the median (line), interquartile range (box) and 5–95 percentile range (whiskers). Compared by one-way ANOVA with Tukey's multiple comparisons. ns, not significant; ***P<0.001. *N*=50 cells, *n*=3. (E) Indicated strains expressing Nvj1–Cherry in High D or D exh. conditions. (F) Quantification of Nvj1–Cherry signal from E. Nvj1 area (in µm²) is depicted as boxplots showing the median (line), interquartile range (box) and 5–95 percentile range (whiskers). Compared by one-way ANOVA with Tukey's multiple comparisons. ns, not significant; ***P<0.001. *N*=50 cells, *n*=3. (G) Snd3–GFP control (Ctrl.) and Snd3–GFP Δ*pex31* cells grown in the indicated conditions. (H) Ratio of Snd3–GFP signal at the NVJ [I_GFP_(NVJ)] to nuclear ER [I_GFP_(nER)] at the opposite side. Box plots show the median (line), interquartile range (box) and minimum to maximum values (whiskers). Compared by one-way ANOVA with Tukey's multiple comparisons. ns, not significant. ***P<0.001. *N*=30 cells, *n*=3. (I) Control (Ctrl.), Δ*pex31*, Δ*snd3* and Δ*snd3*Δ*pex31* cells expressing Nvj1–Cherry were grown in the indicated conditions and subjected to SDS-PAGE and subsequent western blotting. GAPDH is shown as a loading control. (J) Quantification of I. Nvj1–Cherry intensity was normalized to GAPDH intensity and compared to the control sample from D exh. conditions. *n*=3. (K) Cells expressing Pex30–mNG and Nvj1–Cherry in either control (Ctrl.), Δ*pex31*, Δ*snd3* or Δ*snd3*Δ*pex31* background, as indicated. Grown in high glucose. Merge image shows Pex30–mNG in green and Nvj1–Cherry in magenta. (L) Ratio of Pex30–mNG signal at the NVJ [I_GFP_(NVJ)] to cellular mNG signal [I_GFP_(cell)] for cells as in K, depicted as box plots showing the median (line), interquartile range (box) and minimum to maximum values (whiskers). Compared by one-way ANOVA with Tukey's multiple comparisons. ns, not significant; ***P<0.001. *N*=50 cells, *n*=3. (M) Analysis as in (K) with the difference that cells were grown in glucose-exhaustion (D exh.) conditions. (N) Ratio of Pex30–mNG signal at the NVJ [I_GFP_(NVJ)] to cellular mNG signal [I_GFP_(cell)] for cells as in M, depicted as box plots showing the median (line), interquartile range (box) and minimum to maximum values (whiskers). Compared by one-way ANOVA with Tukey's multiple comparisons. ns, not significant; ***P<0.001. *N*=50 cells, *n*=3. Dashed lines in images mark cell boundaries. Scale bars: 5 µm.

Glucose starvation causes not only an expansion of the NVJ proteome (Fig. 1B), but also an increase of the overall NVJ size (Hariri et al., 2018; Roberts et al., 2003; Tosal-Castano et al., 2021). Knowing that *PEX31* deletion partially phenocopies expansion of the NVJ proteome independent of glucose availability (Fig. 2), we asked whether it also affects the size of the NVJ in a similar manner. As an indirect estimate of NVJ size, we quantified the area of Nvj1–Cherry foci at different conditions (Fig. 3A,B; Fig. S3A,B). Consistent with previous reports, we observed an expansion of the Nvj1 area in response to low-glucose conditions in control cells (1.55 µm² mean Nvj1 area at glucose exhaustion versus 1.06 µm² at high glucose) (Fig. 3A,B). Although *PEX31* deletion did not affect Nvj1 area in glucose- or nitrogen-starvation conditions, loss of Pex31 led to a pronounced Nvj1 area enlargement in high-glucose conditions (1.58 µm²). Interestingly, the average Nvj1 area in Δ*pex31* cells in the high-glucose condition was comparable to that of control NVJs in the glucose-exhaustion condition (Fig. 3A,B). No further expansion of the Δ*pex31* Nvj1 area was observed upon glucose exhaustion (Fig. 3A,B), suggesting that NVJs in Δ*pex31* cells have glucose starvation-like features independently of glucose availability.

We assessed further features and functions of the NVJ of Δ*pex31* cells. At glucose restriction, lipid droplets typically accumulate in the region around the NVJ in a manner dependent on the lipid droplet–vacuole tether proteins Ldo16 and Ldo45 (Álvarez-Guerra et al., 2024; Diep et al., 2024). This phenotype was unaffected by *PEX31* deletion (Fig. S3C). Furthermore, Δ*pex31* cells did not have increased lipid droplet accumulation at NVJs in glucose-replete conditions, suggesting that lipid droplet–vacuole tethering was generally unaffected (Fig. S3C). We also assessed PMN, a microautophagy process in which the NVJ is taken up into the vacuolar lumen upon prolonged glucose starvation (Roberts et al., 2003). Consistent with the notion that *PEX31* deletion affects the NVJ specifically in glucose-replete conditions, PMN was comparable between Δ*pex31* and control cells (Fig. S3D,E).

We next created a strain overexpressing *PEX31* from the strong, constitutive *TEF2* promoter. *PEX31*-overexpressing cells showed an Nvj1 area comparable to that of controls in glucose-replete conditions. However, NVJ expansion upon glucose exhaustion was blunted in *PEX31*-overexpressing cells (1.15 µm² mean Nvj1 area in *PEX31*-overexpressing cells versus 1.45 µm² in control cells; Fig. 3C,D). Thus, manipulation of Pex31 levels in either direction affects the NVJ, with loss of Pex31 promoting glucose starvation-like NVJ phenotypes, and high Pex31 levels hampering NVJ adaptation to low glucose. We also assessed NVJ localization of the conditional resident Pex30 in the p*TEF2*-*PEX31* cells, which was, however, unaffected (Fig. S3F,G).

The conditional NVJ resident Snd3 has been identified as an important player in controlling NVJ size. *SND3* deletion causes an overall reduction in NVJ size across metabolic conditions and a block of NVJ expansion upon glucose exhaustion (Tosal-Castano et al., 2021). Consistent with these findings, we observed that *SND3* deletion resulted in a decrease in the Nvj1 area in high-glucose conditions (0.61 µm² average Nvj1 area in Δ*snd3* cells compared to 1.12 µm² in control cells; Fig. 3E,F). When we simultaneously deleted *SND3* and *PEX31*, we found that Δ*pex31*Δ*snd3* double mutant cells had small NVJs (0.55 µm²), comparable to those observed in Δ*snd3* single deletion cells (Fig. 3E,F), showing that Snd3 is required for NVJ expansion in response to loss of Pex31.

Based on the notion that loss of Pex31 promotes accumulation of conditional NVJ proteins, we next asked whether the NVJ expansion observed in Δ*pex31* cells was mediated by recruitment of the NVJ regulator Snd3 to the NVJ. However, while Snd3 accumulated at the NVJ in response to low-glucose conditions as reported previously (Tosal-Castano et al., 2021), Snd3 distribution was not affected by loss of Pex31 (Fig. 3G,H), consistent with the results of our screen for *PEX31*-dependent NVJ residents (Fig. 2B).

Loss of Snd3 has been reported to affect the NVJ by triggering degradation of the main NVJ tether Nvj1 (Tosal-Castano et al., 2021). We therefore assessed Nvj1–Cherry protein levels in Δ*pex31*, Δ*snd3* and Δ*pex31*Δ*snd3* cells in high-glucose and glucose-exhaustion conditions by western blotting. As expected, Nvj1 levels were consistently higher upon glucose exhaustion (Fig. 3I,J). We found that in high-glucose conditions, Nvj1 levels were unaffected by *PEX31* deletion (Fig. 3I,J), showing that NVJ expansion in Δ*pex31* cells in this condition was not mediated by alterations in the amount of this tether protein. Deletion of *SND3*, on the other hand, resulted in reduced Nvj1 levels in both conditions (Fig. 3I,J). This reduction in Nvj1 levels occurred not only in the Δ*snd3* single mutant, but also in Δ*pex31*Δ*snd3* double mutant cells (Fig. 3I,J). In summary, *PEX31* deletion does not promote Snd3 accumulation at the NVJ, suggesting that NVJ expansion in Δ*pex31* cells is not directly driven by Snd3 recruitment. However, deletion of *SND3* leads to a partial loss of the key NVJ tether Nvj1, resulting in a general block of NVJ expansion not only upon glucose exhaustion (Tosal-Castano et al., 2021), but also in response to *PEX31* deletion.

Finally, we asked whether Snd3 is required for remodeling of the NVJ proteome upon *PEX31* deletion. To test this, we assessed localization of Pex30 in Δ*pex31*, Δ*snd3* and Δ*pex31*Δ*snd3* double mutant cells. We found that Pex30 accumulated at the NVJ in Δ*pex31* cells in high glucose conditions both in the presence and in the absence of Snd3 (Fig. 3K,L). This not only shows that Snd3 is dispensable for Pex30 re-localization in Δ*pex31* cells but also suggests that Pex31-dependent modulation of the NVJ proteome does not directly depend on the increase in contact site size, as Pex30 was efficiently recruited to the small NVJs of Δ*pex31*Δ*snd3* double mutant cells. We also analyzed Pex30 distribution upon glucose exhaustion and found that it accumulated at the NVJ as reported previously (Ferreira and Carvalho, 2021) (Fig. 3M,N). Of note, Pex30 was lost from NVJs in Δ*snd3* cells, and this effect was reversed by additional deletion of *PEX31*, further supporting the notion that loss of Pex31 promotes an expansion of the NVJ proteome (Fig. 3M,N).

In summary, loss of Pex31 leads to an increased Nvj1 area in high-glucose conditions, phenocopying the effects of glucose starvation. Both expansion of the NVJ proteome and NVJ enlargement via *PEX31* deletion are not directly mediated via the NVJ regulator Snd3. However, loss of the Nvj1 tether protein upon *SND3* deletion blunts NVJ expansion.

### Δ*pex31* cells show multiple hallmarks of starvation

Loss of Pex31 results in alterations of both NVJ proteome and size that are reminiscent of NVJ features typically observed upon glucose starvation. This prompted us to test whether Δ*pex31* cells display additional hallmarks of glucose starvation related to the vacuole.

**Fig. 4.**
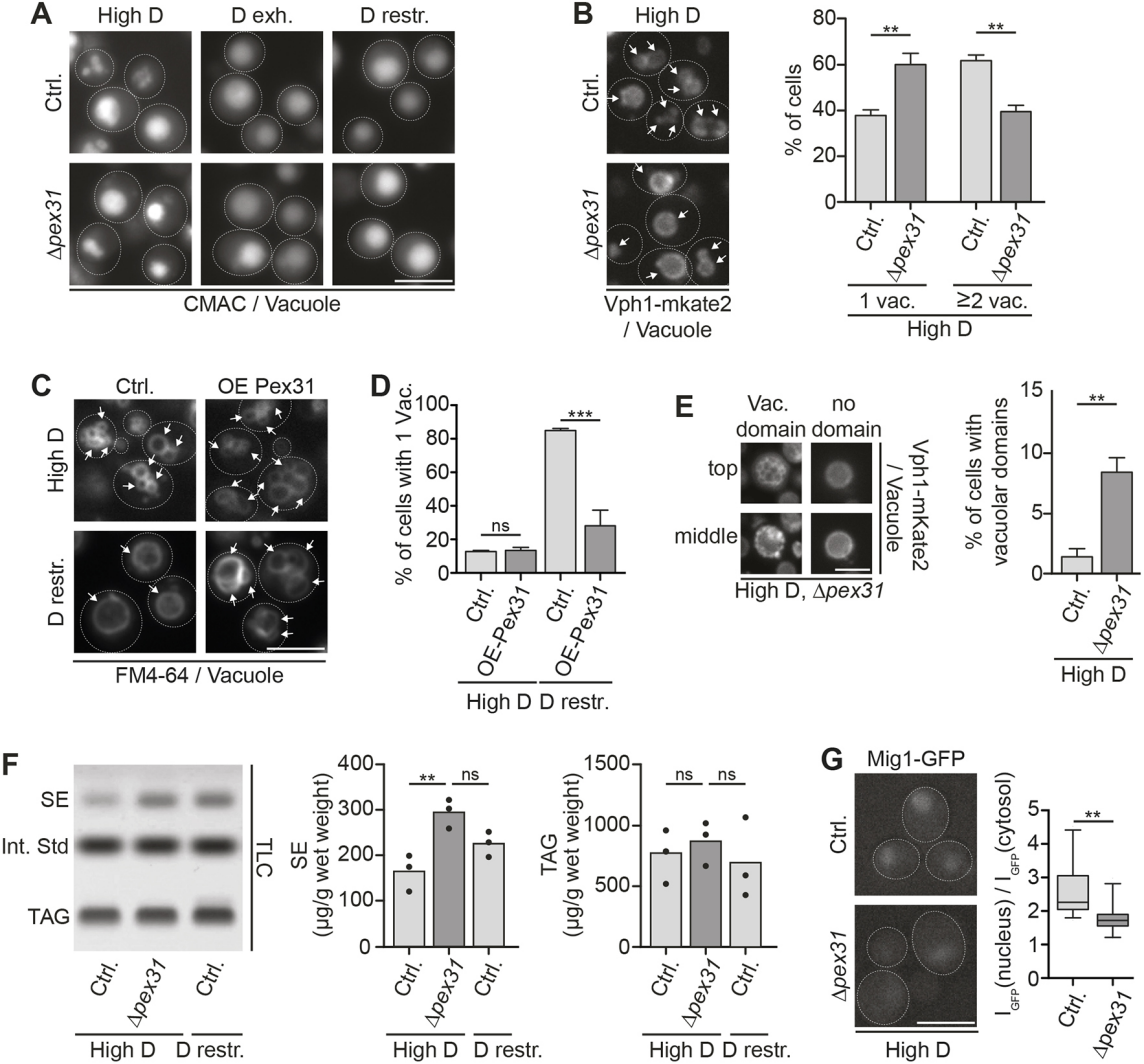
**Absence of Pex31 induces glucose starvation-like phenotypes of lipid storage and vacuolar organization.** (A) Control (Ctrl.) and Δ*pex31* cells grown in the indicated conditions and stained with CMAC for visualization of vacuoles. Images are representative of at least three independent experiments. (B) Left: control and Δ*pex31* cells expressing Vph1–mKate2 as a vacuolar marker in glucose-replete conditions. Arrows indicate the vacuoles. Right: mean percentage of cells showing one vacuole (1 vac.) or at least two vacuoles (≥2 vac.). Error bars show s.e.m. Compared by two-tailed unpaired *t*-test. **P<0.01. *N*=50 cells, *n*=3. (C) Control and *pTEF2*-Pex31 (OE Pex31) cells cultured in the indicated conditions were stained with FM4-64 to visualize the vacuole and analyzed by microscopy. Arrows indicate the vacuoles. (D) Quantification of the percentage of cells with exactly one vacuole from C. Data are presented as mean±s.e.m. Compared by two-tailed unpaired *t*-test. ns, not significant; ***P<0.001. *N*=50 cells, *n*=3. (E) Left: example images of Δ*pex31* cells with vacuolar domains (Vac. domain) and without (no domain). *Z*-stacks of Vph1–mKate2-expressing cells were analyzed, and images from the top and middle of representative *z*-stacks are shown. Right: percentage of cells with vacuolar domains in glucose-replete conditions. Data represented as mean±s.e.m. Compared by two-tailed unpaired *t*-test. **P<0.01. *N*=50 cells, *n*=3. (F) Left: HPTLC plate of lipid extracts from control and Δ*pex31* cells in high-glucose (High D) or glucose-restriction (D restr.) conditions showing neutral lipid separation. SE, sterol ester; TAG, triacylglycerol; Int. Std, internal standard (cholesteryl formate). Right: quantification of sterol ester and triacylglycerol in (µg/g wet weight). Compared by one-way ANOVA with Tukey's multiple comparisons. ns, not significant; **P<0.01. *n*=3. (G) Left: control and Δ*pex31* cells expressing Mig1–GFP grown in glucose-replete (High D) conditions. Right: quantification of Mig1–GFP intensity in the nucleus [I_GFP_(nucleus)] in comparison to cytosolic GFP intensity [I_GFP_(cytosol)]. Data are shown in boxplots depicting the median (line), interquartile range (box) and 5–95 percentile range (whiskers). Compared by two-tailed unpaired *t*-test. **P<0.01. *N*=50 cells, *n*=3. Dashed lines in images mark cell boundaries. Scale bars: 5 µm.

A characteristic feature of glucose-exhausted or glucose-restricted cells is an alteration in vacuolar morphology. While cells often have multilobed vacuoles in high-glucose conditions, low glucose typically promotes formation of a single large, spherical vacuole (Armstrong, 2010; Li and Kane, 2009). To assess vacuolar morphology, we stained control and Δ*pex31* cells in glucose-replete, -restricted and -exhausted conditions with the vacuolar lumen dye CMAC. As expected, control cells showed a mixed phenotype, with both multilobed and single vacuoles in glucose-replete conditions, whereas vacuoles typically fused into one large structure in low-glucose conditions (Fig. 4A). In contrast, Δ*pex31* cells often showed large single vacuoles independently of glucose availability, phenotypically mimicking low-glucose conditions in the presence of high glucose (Fig. 4A). To quantify this effect, the vacuolar membrane marker Vph1–mKate2 was expressed in control and Δ*pex31* cells to optimally resolve single vacuoles. Quantification of cells with one vacuole versus cells with two or more vacuoles in high-glucose conditions confirmed a pronounced shift toward single large vacuoles in the Δ*pex31* cells (Fig. 4B).

We next performed the inverse experiment and assessed vacuole morphology in *PEX31*-overexpressing cells. At glucose repletion, both *PEX31*-overexpressing cells and control cells contained mainly multilobed vacuoles. However, upon glucose restriction, *PEX31* overexpression blocked the shift to the typical phenotype of single, large vacuoles that was observed in control cells (Fig. 4C,D).

Glucose exhaustion has also been found to promote a lateral reorganization of the vacuolar membrane. Liquid-ordered (likely sterol-rich) and liquid-disordered membrane domains are formed, and this process can be visualized via Vph1–mKate2, which specifically segregates into the liquid-disordered membrane regions (Toulmay and Prinz, 2013; Wang et al., 2014). Consistent with previous observations, we found that these domains were very rare at glucose repletion in control cells (∼1% of cells). In contrast, ∼8% of Δ*pex31* cells showed vacuolar membrane domains at glucose repletion (Fig. 4E). In conclusion, *v*acuoles of Δ*pex31* cells in high-glucose conditions show multiple features of glucose starvation.

Next, we asked whether Δ*pex31*-dependent alterations had consequences on NVJ functions in lipid metabolism and storage. It has been found that accumulation of the HMG-CoA reductases Hmg1 and Hmg2 at the NVJ during glucose restriction promotes flux through the mevalonate pathway and sterol ester synthesis (Rogers et al., 2021). We found that *PEX31* deletion drives not only glucose-independent accumulation of Hmg2 at the NVJ, but also a similar re-localization of the Hmg2 stabilizer Nsg1 (Fig. 2B,E,F). To test whether this Pex31-dependent reorganization reflects on the lipidome of the cell, we used high-performance thin-layer chromatography (HPTLC) to analyze the neutral lipid content of control and Δ*pex31* cells in glucose-replete conditions. We found that *PEX31* deletion indeed resulted in an increase in sterol ester levels at glucose repletion, whereas levels of triglycerides were unaffected (Fig. 4F). Similar to our findings on the size (Fig. 3) and composition (Fig. 2) of the NVJ, sterol ester levels in Δ*pex31* cells in glucose-replete conditions were comparable to those observed in glucose-restricted control cells (Fig. 4F). We tested whether these alterations in sterol ester levels reflected on the number of lipid droplets formed, but we did not detect differences (Fig. S4A,B), presumably due to the high amount of stored triacylglycerol, the second main lipid droplet core component. Growth of Δ*pex31* cells was comparable to that of controls at different glucose levels (Fig. S4C). We next asked whether the observed alterations in sterol ester storage in Δ*pex31* cells can be directly linked to alterations in Hmg2 localization. We created Δ*hmg2*Δ*pex31* cells and analyzed their neutral lipid content. Indeed, *HMG2* deletion resulted in a drastic drop in sterol ester levels both in the control and the Δ*pex31* background (Fig. S4D). We noted a minor, statistically non-significant elevation in sterol ester levels in Δ*hmg2*Δ*pex31* cells over those in Δ*hmg2* cells, indicating that additional factors besides Hmg2 re-localization could be involved.

Finally, we analyzed cellular distribution of Mig1, a transcriptional repressor involved in glucose repression (Nehlin and Ronne, 1990). Mig1 localization responds to glucose availability, with glucose promoting its accumulation in the nucleus for transcriptional repression (Vit et al., 1997). Mig1 levels were comparable between control and Δ*pex31* cells (Fig. S4E). Mig1 accumulated in the nucleus of control cells in glucose-replete conditions, as expected. In contrast, this effect was decreased in Δ*pex31* cells (Fig. 4G), pointing toward an alteration in glucose-dependent transcriptional repression in Δ*pex31* cells.

In summary, loss of Pex31 provokes not only a reorganization of the NVJ reminiscent of alterations that typically occur in low-glucose conditions, but also further glucose starvation-related adaptations of vacuole remodeling, neutral lipid storage and, potentially, transcriptional control.

## DISCUSSION

Here, we have used microscopy-based high-content screens to characterize the NVJ in glucose-replete and glucose-starved conditions and have identified permanent and condition-specific contact site residents. We find that one of these residents, Pex31, has a role in metabolic adaptation of the size and composition of the NVJ. The contact site generally has a limited size and proteome in high-glucose conditions. Upon glucose starvation, the NVJ size increases and the contact site becomes populated by additional residents. Intriguingly, Pex31 shows the inverse behavior, being enriched at the small NVJ structures observed at glucose repletion and being lost from the contact upon starvation. Furthermore, *PEX31* deletion uncouples NVJ expansion from metabolic cues: in Δ*pex31* cells, the Nvj1 area expands in high-glucose conditions, comparable to the size typically observed during glucose starvation. At the same time, a set of normally glucose-starvation-specific contact site residents populate the Δ*pex31* NVJ irrespective of high glucose availability. Overall, these findings are consistent with a model where Pex31 has a role in restricting NVJ size and composition when glucose is replete.

We assessed the relationship of Pex31 to Snd3, another NVJ modulator. Deletion of *SND3* has been shown to result in destabilization of the key NVJ tether Nvj1 and a block in NVJ expansion in response to glucose starvation (Tosal-Castano et al., 2021). We find that loss of Snd3 and the concomitant decrease in Nvj1 tether levels broadly blunts NVJ size expansion, not only upon glucose starvation but also in Δ*pex31* cells. However, expansion of the NVJ proteome in Δ*pex31* cells was unaffected by *SND3* deletion, showing that NVJ remodeling by Pex31 is not directly mediated by Snd3.

Pex31 is part of a structurally related protein family that, besides Pex31 itself, comprises Pex28, Pex29, Pex30 and Pex32 (referred to hereafter as Pex28/29/30/31/32 proteins). Although the exact molecular roles of the Pex28/29/30/31/32 proteins are unclear, it is emerging that their functions are related to the maintenance of ER membrane homeostasis. All family members are ER-integral proteins anchored in the membrane by an N-terminal RHD (Joshi et al., 2016), which is similar to the RHDs of the membrane-shaping reticulon proteins (Voeltz et al., 2006). Indeed, Pex30 and Pex31 overexpression has been found to restore viability of a mutant simultaneously lacking the reticulons Rtn1 and Rtn2, the reticulon-binding protein Yop1 and the lipid metabolism regulator Spo7, an effect that has been suggested to be linked to the ER membrane-shaping ability of the Pex30 and Pex31 RHDs (Joshi et al., 2016). C-terminal to the RHD, all members of the family contain an additional dysferlin domain. These domains bind to phosphatidic acid *in vitro*, and loss of Pex28/29/30/31/32 proteins results in aberrant phosphatidic acid distribution within the ER membrane (Ferreira et al., 2025; House et al., 2025). All Pex28/29/30/31/32 proteins affect formation of ER-derived organelles. Individual loss of each family member results in altered abundance and morphology of peroxisomes (Vizeacoumar et al., 2003, 2004). Pex30 has been found to have a role in the organization of specialized ER membrane subdomains and in the formation of pre-peroxisomal vesicles from these domains (Joshi et al., 2016). Additionally, Pex30 is involved in formation of lipid droplets from these ER membrane regions (Choudhary et al., 2020; House et al., 2025; Joshi et al., 2018; Wang et al., 2018). Immunoprecipitation has revealed that Pex30 forms different heterooligomeric complexes with other family members that localize to distinct organelle contact sites: Pex28–Pex30–Pex32 complexes that reside at ER–peroxisome contacts, and Pex29–Pex30 complexes, which localize to the NVJ upon glucose starvation (Ferreira and Carvalho, 2021). Intriguingly, the same study revealed that Pex31 is uniquely excluded from complex formation with Pex30 (Ferreira and Carvalho, 2021), raising questions about the functional relation of this protein. We find that Pex31 is also an NVJ resident; however, its response to metabolic states and its effect on the contact site are inverse. Whereas Pex29–Pex30 complexes localize to the NVJ specifically upon glucose starvation, Pex31 is enriched at this contact site in glucose-replete conditions. Furthermore, whereas Pex29 and Pex30 are required for NVJ expansion (Ferreira and Carvalho, 2021; Ferreira et al., 2025), we find that Pex31 counteracts the expansion of the NVJ size and proteome, suggesting antagonistic effects of Pex29–Pex30 and Pex31. Interestingly, *PEX31* deletion not only phenocopies a state of starvation in terms of NVJ organization. Instead, further hallmarks of glucose deprivation are induced, including a shift from multilobed to single, round vacuoles, a reorganization of the vacuole membrane into liquid-ordered and liquid-disordered domains, an increase in the storage of sterol esters, and a reduced nucleoplasmic accumulation of the transcriptional repressor Mig1, a central player in glucose repression. The exact mechanistic basis of this starvation-like organelle reprogramming via loss of Pex31 is an important topic for future studies.

More broadly, we have expanded our view on the proteome of the NVJ, offering future directions for the analysis of this central organelle interface. The finding that the Hmg2-stabilizing proteins Nsg1 and Nsg2 accumulate at the NVJ upon glucose starvation opens interesting directions for the regulation of sterol metabolism. Nsg1 and Nsg2 are the yeast homologs of the mammalian INSIG proteins (Sever et al., 2003; Yang et al., 2002). The Nsg proteins stabilize Hmg2 by preventing its degradation via ER-associated degradation (Flury et al., 2005). We find that not only glucose starvation but also manipulation of the NVJ by loss of Pex31 induce a re-localization of both Hmg2 and Nsg1 to the NVJ. The details of how this mechanistically relates to the increased sterol ester accumulation that we detect in Δ*pex31* cells remain to be determined. Of note, a study submitted in parallel to this work also identified Nsg1 and Nsg2 as glucose starvation-specific NVJ residents and explored their localization and function in more detail (Fujimoto and Tamura, 2025 preprint). We also detect Shr5 at the NVJ, a subunit of a palmitoyl transferase that together with Erf2 palmitoylates Ras nutrient signaling GTPases, regulating their membrane localization and activation (Dong et al., 2003; Jung et al., 1995; Lobo et al., 2002; Zhao et al., 2002). Investigating the role of Shr5 at the NVJ and potential implications for Ras signaling is an interesting topic for the future. Finally, we detect Tcb1 at the NVJ upon glucose starvation. Tcb1 is a member of the Tcb1/2/3 tricalbin family and a well-characterized ER–plasma membrane contact site resident that acts as a tether and lipid transfer protein (Collado et al., 2019; Creutz et al., 2004; Hoffmann et al., 2019; Manford et al., 2012; Toulmay and Prinz, 2012). Interestingly, *NVJ1* shows negative genetic interactions with the tricalbins (Hoffmann et al., 2019). This suggests partially redundant functions of ER–plasma membrane contacts and the NVJ. Alternatively, the finding that Tcb1 can re-localize to the NVJ opens the possibility for a more direct mechanism underlying this genetic link. The exact role of Tcb1 at the NVJ remains elusive, but it is tempting to speculate that the dual localization to ER contact sites with the plasma membrane and the vacuole might serve a regulatory role. Similar dual localizations to distinct contact sites have been described, for example, for Vps13 (Lang et al., 2015), Nvj2 (Liu et al., 2017; Toulmay and Prinz, 2012), Osh1 (Elbaz-Alon et al., 2015; Gatta et al., 2015; Kvam and Goldfarb, 2004; Levine and Munro, 2001) and members of the Lam protein family (Elbaz-Alon et al., 2015; Gatta et al., 2015; Murley et al., 2015).

In conclusion, we have in this work identified multiple additional NVJ residents and a regulator of proteomic NVJ adaptation to metabolic cues, opening new directions toward a mechanistic understanding of the functional plasticity of this intriguing organelle interface.

## MATERIALS AND METHODS

### Yeast strains and growth conditions

*Saccharomyces cerevisiae* strains used in this study were derived from a synthetic genetic array (SGA) compatible strain (Breslow et al., 2008) and are described in Table S2. Cells were transformed with PCR products using a method including lithium acetate, polyethylene glycol and single-stranded DNA (Gietz and Woods, 2006; Longtine et al., 1998). Plasmids were created using Gibson assembly (Gibson et al., 2009, 2010). For this, PCR fragments were mixed with the NEBuilder HIFi DNA Assemly Master Mix (NEB) and incubated at 50°C for 15 min. Primers for genetic modifications and validation were designed using Primers-4-Yeast (Yofe and Schuldiner, 2014). Plasmids and primers used are listed in Tables S3 and S4.

Yeast cells were grown overnight as a pre-culture in synthetic medium (0.67% weight/volume yeast nitrogen base with ammonium sulfate, 2% weight/volume glucose, amino acid supplements, adenine hemisulfate) at 30°C, 280 rpm. Cells in glucose exhaustion (D exh.) were kept undiluted growing into stationary phase for 20–24 h. For glucose-replete conditions (High D), cells were back-diluted from the overnight culture and grown for 4 h until reaching logarithmic growth in synthetic medium with 2% glucose. Glucose-restricted cells (D restr.) were collected from the overnight culture, washed twice with synthetic medium containing 0.001% glucose and grown for 4 h in this medium. For nitrogen starvation, cells were back diluted in synthetic medium without nitrogen with 2% glucose for 24 h. For induction of PMN, cells were grown overnight, diluted 1:100 and further grown for 24, 48 or 72 h respectively.

### Fluorescence microscopy

Cells were transferred to a 384-well glass-bottom plate (Brooks) coated with Concanavalin A (Sigma-Aldrich). After 15 min of incubation, the medium was replaced. Images were acquired either with an Olympus IX83 inverted fluorescence microscope with a Lumencor SpectraX LED light source and a 40× or 60× air objective using the Olympus ScanR Automated Image Acquisition Software or with an Opera Perkin Elmer microscope with a laser light source and a 60× water immersion objective (NA=1.2) using the Opera Software 2.0 (EvoShell). For Fig. 1C–E cells were imaged using an iMIC-based microscope (FEI /Till Photonics) with an Olympus 100× oil objective (NA 1.45) and a laser light source. For Fig. S2A, cells were imaged using the Zeiss LSM980 Airyscan 2 microscope and processed using Joint Deconvolution. For staining of the vacuole, either CellTracker Blue CMAC dye (7-amino-4-chloromethylcoumarin; 0.1 mM; ThermoFisher) was added to the cells for 30 min or cells were stained with FM4-64 (8 µM; Thermo Fisher) during their back-dilution. Subsequently, the medium was replaced and cells were imaged. For visualization of lipid droplets, cells were stained with 1 µM BODIPY 493/503 (Sigma-Aldrich) for 15 min.

### Library generation by automated mating and microscopy-based screening

The query strains Nvj1–Cherry, Zrc1–Cherry and *pex31* were constructed using a synthetic genetic array (SGA) compatible strain (Breslow et al., 2008) and crossed with a collection of mutants selected from the genome-wide SWAT collection or with the full SWAT collection (Weill et al., 2018; Yofe et al., 2016) using automated mating (Cohen and Schuldiner, 2011; Tong and Boone, 2006; Yofe et al., 2016). Automated mating was performed using the RoToR benchtop colony array instrument (Singer instruments). Strains were mated on YPD medium [1% yeast extract (Thermo Fisher, 212750), 2% Bacto peptone (Thermo Fisher, 211677), 2% glucose (Sigma-Aldrich, G8270), 2.2% agar (Becton Dickinson, 21030)] and selected for diploids before sporulation was induced by transfer to nitrogen-starvation medium for 5 days. To select for haploids, cells were moved to plates containing 50 mg/l canavanine (Sigma-Aldrich, C9758) and 50 mg/l thialysine (Sigma-Aldrich, A2636). For the final selection step, cells were transferred to a plate containing selections for all desired mutations. For imaging, the strains were grown in 384-well polystyrene plates in synthetic medium in the indicated growth conditions. Cells were transferred to 384-well glass plates (Brooks) using the Bench Smart 96 liquid handler (Mettler Toledo).

### Growth assay

Cells were grown to logarithmic growth phase (High D) and back-diluted to an optical density at 600 nm (OD_600_) of 0.05. From this sample a 10× dilution series was prepared, and 5 µl of each dilution was spotted on either YP plates [1% yeast extract (Thermo Fisher, 212750), 2% Bacto peptone (Thermo Fisher, 211677), 2% agar (Becton Dickinson, 21030)] with the indicated glucose (Sigma-Aldrich, G8270) concentrations or synthetic complete (SC) plates with the indicated glucose concentrations. The plates were incubated at 30°C for 48 h.

### Whole-cell extraction, SDS-PAGE and western blotting

For the western blots, 2.5 OD_600_ of cells in glucose-replete (High D), glucose-exhausted (D exh.) or glucose-restriction (D restr.) conditions were harvested by centrifugation and resuspended in urea lysis buffer (8 M urea, 50 mM Tris-HCl, pH 7.5) with protease cocktail (1:200, Merck). Acid-washed glass beads were added, and the samples were vortexed at maximal velocity for 4×5 min, with 1 min pause on ice in between. The supernatant was collected, and a fraction was used to measure protein concentration by a Bradford assay. Then, samples were incubated for 5 min at 95°C in MSB ox. buffer [8 M urea, 0.15% (w/v) Bromophenol Blue, 5 mM EDTA, 3.2% SDS, 100 mM Tris-HCl, 4% (v/v) glycerol, 10% β-mercaptoethanol]. Samples of cell extractions were subjected to SDS-PAGE (4–15% Mini-PROTEAN TGX Precast Protein Gel, Bio-Rad) and subsequent western blotting. Antibodies used are listed in Table S5. Signals were detected using the Lumi-Light^PLUS^ substrate (Roche) and the Azure 600 imaging system. Quantification of protein levels in Fig. 3J was performed with the AzureSpot software. Intensity of each band was measured and normalized to the respective GAPDH intensity. Ratio of Nvj1–Cherry compared to the control are depicted. A blot transparency figure is included in the supplementary information (Fig. S5).

### High-performance thin-layer chromatography

Overnight cultures were grown in synthetic medium containing 2% glucose. For the logarithmic growth phase (High D), the same medium was used to inoculate the main cultures with an OD600 of 0.2. Cells were incubated at 30°C and shaken at 160 rpm until reaching an OD600 of 0.8. For glucose restriction, an adequate amount of cells was collected from the overnight culture and washed twice with synthetic medium containing 0.001% glucose. The main culture, using the low-glucose medium, was inoculated at an OD600 of 0.8. Cells were shaken at 160 rpm and 30°C for 4 h.

For lipid extraction, a total of 180 mg (wet weight) was harvested of each culture. The protocol for total lipid extraction was based on Folch et al. (1957). 5 ml of a chloroform/methanol (CHCl_3_/MeOH) 2:1 (v/v) mixture and 1 ml of glass beads (Sigma-Aldrich, G8722), together with 125 µg of cholesteryl formate (Sigma Aldrich, S44853) as internal standard was added to each cell pellet. For lysis the mixture was shaken using a Heidolph Multi Reax shaker for 30 min at level 8. After addition of 1 ml H_2_O, shaking was continued for another 10 min. The samples were centrifuged for 5 min at 2500 ***g***, which resulted in formation of two phases. The aqueous phase was carefully discarded, and 2 ml of an artificial upper phase comprising MeOH/H_2_O/CHCl_3_ (48/47/3; v/v/v) was added. Following a short mixing, samples were again centrifuged (5 min, 2500 ***g***), the aqueous phase discarded and the organic phase collected. A nitrogen stream was used to completely evaporate the solvent, and the dried samples were subsequently dissolved in 1 ml CHCl_3_/MeOH (2:1; v/v) and stored at −20°C.

For quantification of neutral lipid contents, between 30 µl and 100 µl of total lipid extracts was applied to an HPTLC silica gel 60 plate (20×10 cm) using a CAMAG automatic TLC sampler (CAMGA, ATS4). A mobile phase consisting of *n*-hexane/*n*-heptane/diethyl ether/acetic acid (63/18.5/18.5/1; v/v/v/v) was used to separate neutral lipids. This developing step was performed by the CAMAG automatic developing chamber (ADC2). To visualize the separated lipids, the plate was derivatized with 0.01% primuline (Sigma-Aldrich, 206865) using the CAMAG derivatizer and afterwards heated to 40°C for 2 min on the CAMAG TLC plate heater 3. Image acquisition of the fully developed HPTLC plates was executed by the CAMAG TLC visualizer 2 using the VisionCATS software. To determine the different lipid concentrations, the HPTLC bands were converted into chromatograms and evaluated via a standard curve. This standard curve was generated by applying a neutral lipid standard mix together with the samples on the HPTLC plate in quantities ranging from 0.5 µg to 15 µg lipid. The standard mix comprised triacylglycerol (15:0-18:1-15:0; Sigma-Aldrich, 330723C), diacylglycerol (18:1; Sigma-Aldrich, 800811C), sodium oleate (Sigma-Aldrich, O7501), ergosterol (Thermo Fisher Scientific, 117810050), cholesteryl oleate (18:1; Sigma-Aldrich,700269P) and cholesteryl formate (Sigma-Aldrich, S448532) dissolved in CHCl_3_/MeOH (2:1; v/v), each at a concentration of 500 ng/µl. The neutral lipid contents were standardized according to the internal standard.

### Quantifications and statistical analysis

All microscopic images were processed using Fiji/ImageJ. Line graphs were generated using Fiji/ImageJ to generate an intensity profile after background subtraction. The intensity profiles were normalized to the maximum value and plotted using GraphPad Prism 6.

For Pex31 localization in Fig. 1F, cells were divided into three classes: (1) Pex31 signal that completely or partially colocalizes with Nvj1–Cherry, (2) Pex31 foci that are at the nuclear ER but not overlapping with Nvj1–Cherry signal and (3) cells that do not show any Pex31 foci at the nuclear ER. Percentage of each class is depicted.

For quantification of enrichment of signal at the NVJ, a first region of interest (ROI) was generated at the area defined as the NVJ. A second ROI was drawn either around the cell (Fig. 2C,D), on the opposite side of the nuclear ER (Fig. 2E,F) or in the cytosol (Fig. 2G). The ratio between the mean signal at the NVJ versus the control ROI is depicted. For measurement of the Nvj1 area (Fig. 3B,D,F), ROIs of the cells to be analyzed were generated and overlayed on a binary image in which the NVJ was selected using the Otsu threshold. The size of the NVJ was then measured using the Analyze Particle function of ImageJ. For vacuolar fragmentation (Fig. 4B,D), vacuoles in each cell were counted, and cells were categorized into cells containing either one vacuole (1 vac.) or two or more vacuoles (≥2 vac.). For vacuolar domains, *z*-stacks of Vph1–mKate2-expressing cells were imaged (Fig. 4E). Based on the *z*-stacks, each cell was categorized dependent on vacuolar domain content.

To determine the number of lipid droplets per cell (Fig. S4B), the Find Maxima option in ImageJ was used to highlight lipid droplets, which were counted. For Mig1–GFP analysis, GFP intensity in the nucleus versus GFP intensity in the cytosol was measured after background subtraction (Fig. 4G). The ratio of the mean GFP intensity in the nucleus versus mean GFP intensity in the cytosol was calculated.

For visualization and statistical comparison Graph Pad Prism 6 was used. The bar graphs in Figs 3J and 4F were generated using Graph Pad Prism 10. Data were obtained from at least three independent experiments. The number of cells analyzed is indicated in the respective figure legends. The whiskers of box plots are indicated in the figure legends. For area measurement, the whiskers depict 5–95% of the data. For comparison between two strains a *t*-test was used (Figs 2C–G and 4B,D,E,G; Fig. S3B,E). Comparison of multiple samples was performed using one-way ANOVA with Tukey's multiple comparisons (Fig. 3B,D,F,H,L,N; Figs S3G, S4B) or Bonferroni (Fig. 4F; Fig. S4D). Statistical significance was defined as *P<0.05; **P<0.01; ***P<0.001.

## Supplementary Material

10.1242/joces.264171_sup1 Supplementary information

Table S1. Related to Fig. 1. List of genes analyzed in microscopy-based screen for NVJ resident proteins.
