# Supplementary material for: Role of Pex31 in metabolic adaptation of the nucleus–vacuole junction
Source: J Cell Sci. 2025 Nov 21;138(22):jcs264171. doi: 10.1242/jcs.264171 (PMC12669968; doi:10.1242/jcs.264171)
Supplement: Supplementary information [file joces-138-264171-s1.pdf]

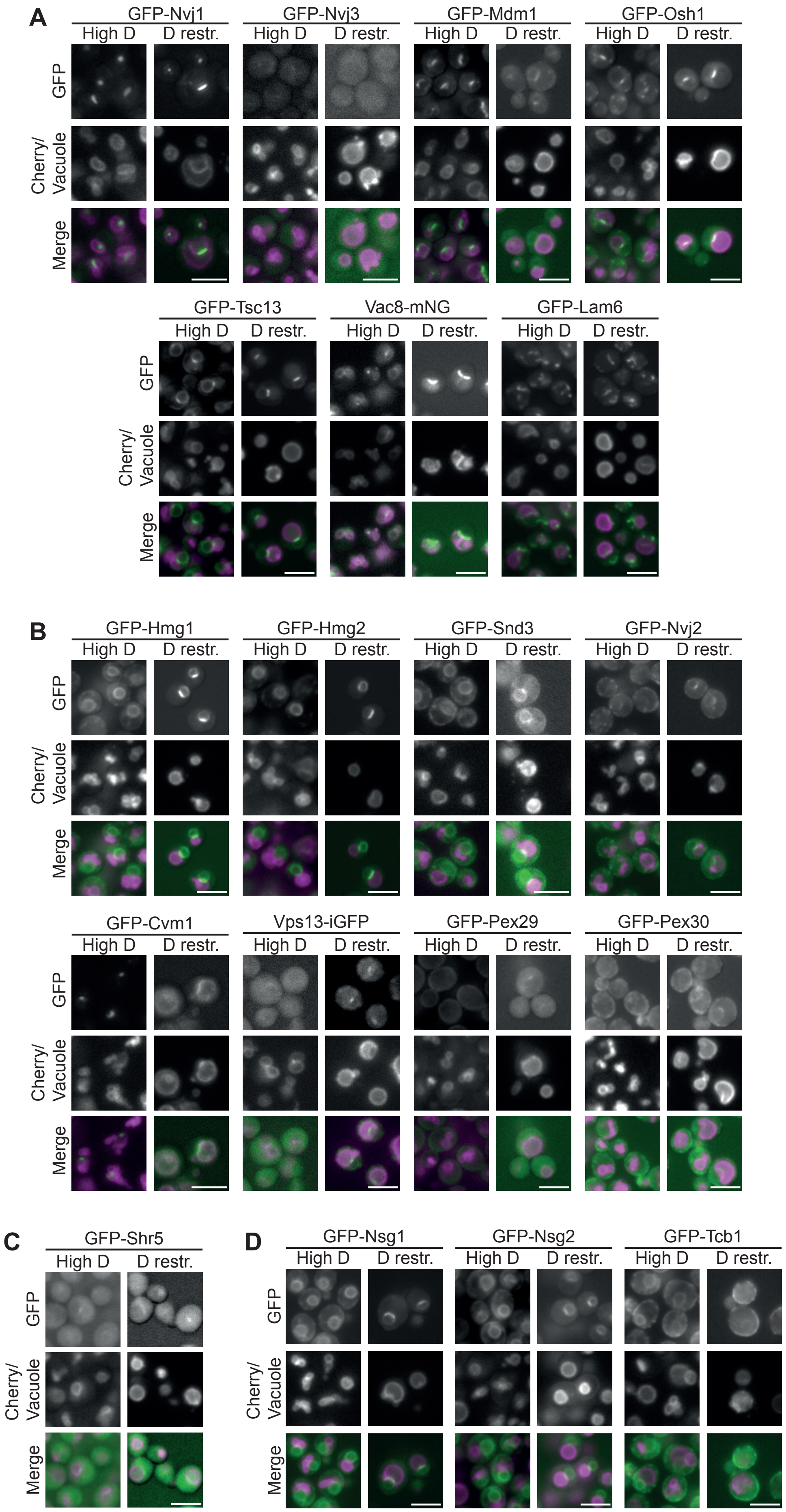

Journal of Cell Science • Supplementary information

Fig. S1. Related to Fig. 1. A microscopy-based screen identifies permanent and conditional residents of the nucleus vacuole junction NVJ.

(A)-(D) Original images of hits identified in screen described in Fig. 1A and B. Using automated mating, either Nvj1-Cherry or Zrc1-Cherry were introduced into a collection of strains expressing proteins related to lipid handling fused with green fluorescent tags. Strains were cultured overnight on medium containing 2 % glucose and either back diluted in fresh medium containing 2 % glucose and grown to logarithmic growth phase (High D), or cells were back diluted in medium containing 0.001 % glucose and incubated for four hours (D restr.). All strains were analyzed by automated microscopy and classified as permanent (A and C) or conditional (B and D) NVJ residents. Scale bars, 5 μm.

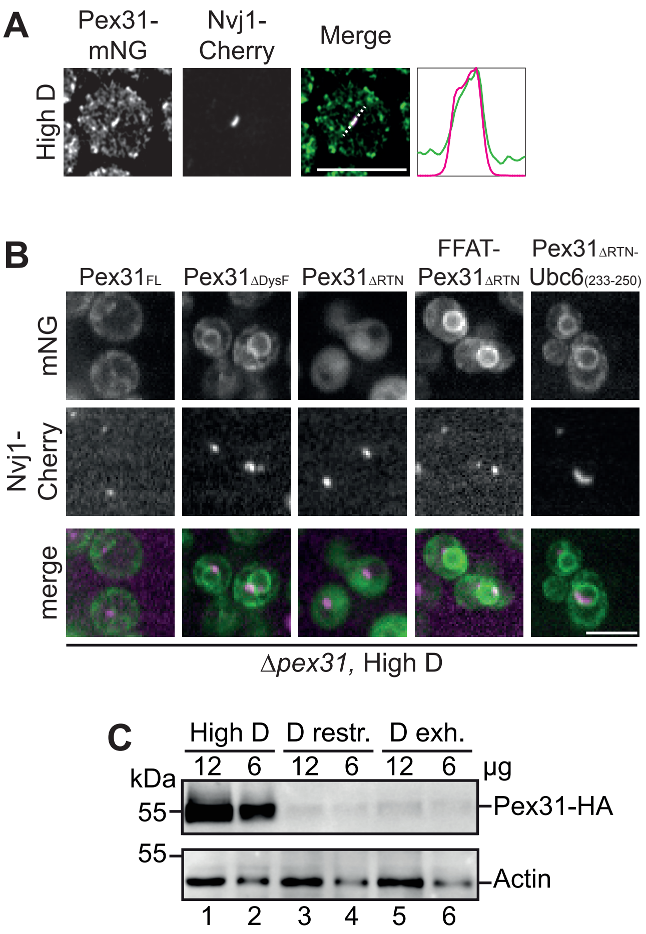

Fig. S2. Related to Fig. 1. Characterization of Pex31.

(A) Pex31-mNG and Nvj1-Cherry co-expressing cells were grown at glucose replete conditions and analysed by Airyscan laser scanning microscopy. Scale bar, 5 µm.

(B) Δ*pex31* cells expressing Nvj1-Cherry and indicated Pex31-mNG variants were grown to logarithmic growth phase (High D) and analyzed by microscopy. Scale bar, 5 µm.

(C) Pex31-HA cells were cultured at indicated conditions and subjected to SDS-PAGE and western blotting.

Journal of Cell Science • Supplementary information

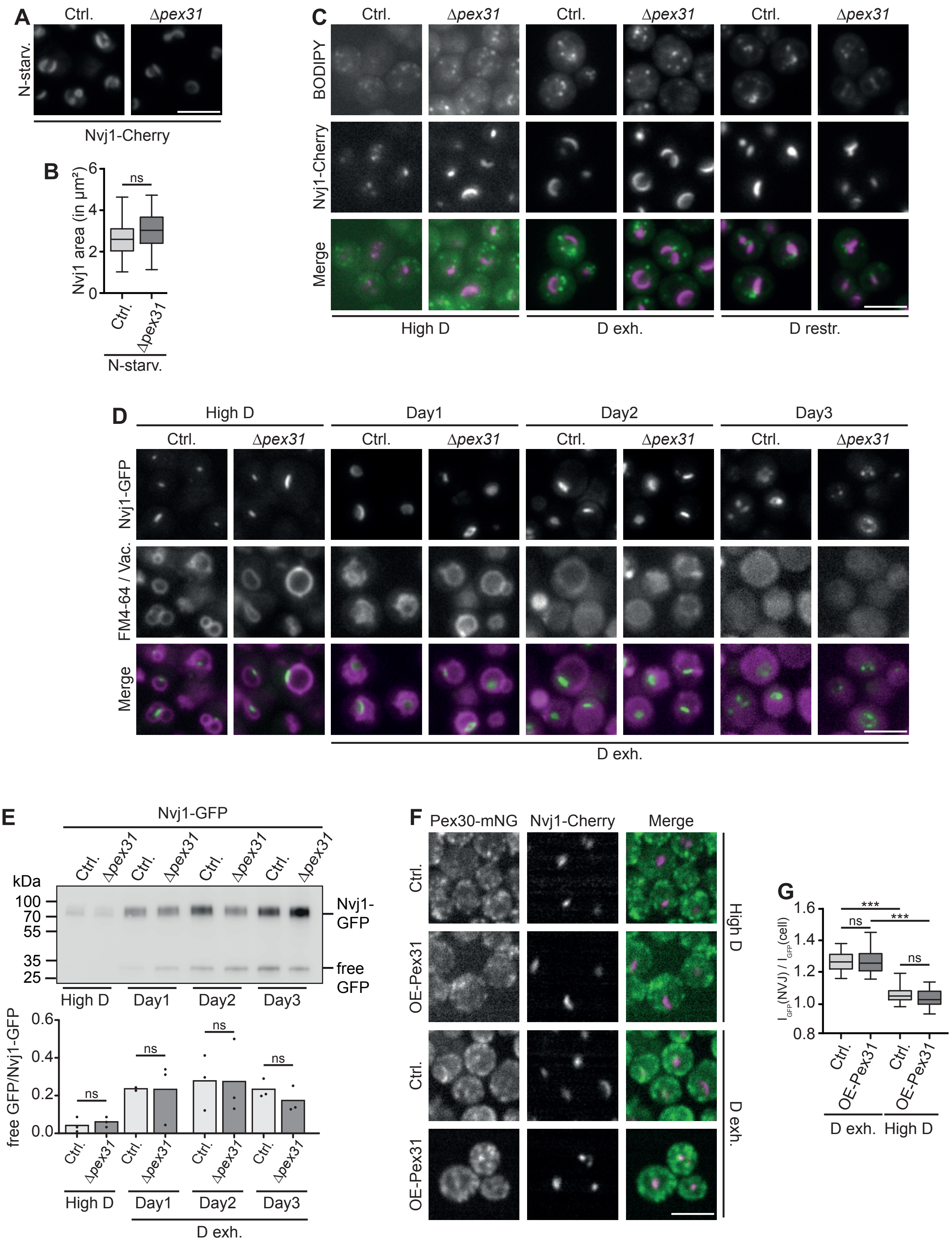

Journal of Cell Science • Supplementary information

Fig. S3. Related to Fig. 3. Analysis of lipid storage and PMN in Δ*pex31* and *PEX31* overexpressing cells.

(A) Control and Δ*pex31* cells expressing Nvj1-Cherry were cultured at nitrogen starvation conditions and analysed by microscopy. Scale bar, 5 µm.

(B) Quantification of Nvj1-Cherry signal from (A). Nvj1 area in μm^2 is depicted as boxplots (5-95 percentile). Compared by t-test. ns, not significant. N=50 cells, n=3.

(C) Control and Δ*pex31* cells expressing Nvj1-Cherry were grown at indicated conditions and analysed by microscopy. Scale bar, 5 µm

(D) Control and Δ*pex31* cells expressing Nvj1-GFP were grown into glucose exhaustion as indicated, stained with FM4-64 to label vacuoles (Vac.) and analysed by microscopy. Scale bar, 5 µm.

(E) Top: Cells analysed in (D) were subjected to SDS-PAGE and subsequent western blotting. Bottom: Ratio of free-GFP intensity compared to full length Nvj1-GFP was determined as a measure of piecemeal autophagy of the nucleus. Compared by t-test. ns, not significant. n=3.

(F) Control and Pex31 overexpressing (OE) cells expressing Pex30-mNG and Nvj1-Cherry were grown in indicated conditions. Scale bar, 5 µm.

(G) Enrichment of GFP signal from (F) at the NVJ (I_{GFP}(NVJ)) over the whole cell (I_{GFP}(cell)) was quantified. Data is shown in boxplots depicting the median with whiskers showing the minimum and maximum value. Compared by one-way ANOVA. ns, not significant; ***, $p < 0.001$. N=30 cells, n=3.

Journal of Cell Science • Supplementary information

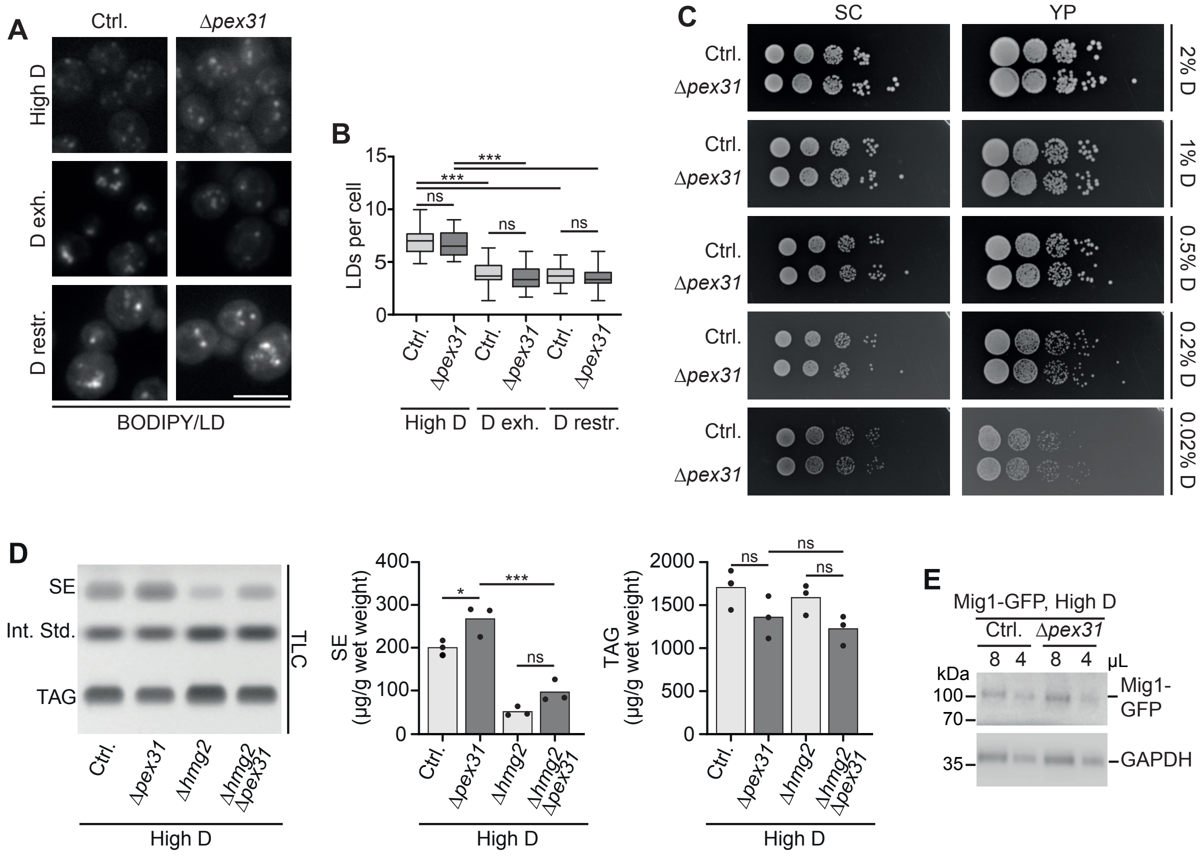

Fig. S4. Related to Fig. 4. Characterization of glucose starvation-related phenotypes in Δ*pex31* cells.

(A) Control and Δ*pex31* cells were grown at indicated conditions and stained with the neutral lipid dye BODIPY493/503 to visualize lipid droplets. Scale bar, 5 μm.

(B) Quantification of lipid droplet abundance in samples shown in (A). Box plots depict median and 5-95 percentile. Compared by one-way ANOVA. ns, not significant; ***, $p < 0.001$. N=50 cells, n=3.

(C) Growth assessment of control and Δ*pex31* cells. Cells were spotted in a 10x dilution series on agar plates containing indicated media. SC, synthetic complete; YP, yeast extract and peptone.

(D) Left: High performance thin layer chromatography (HPTLC) plate of lipid extracts from indicated strains cultured to logarithmic growth phase on medium containing 2% glucose (High D) showing neutral lipid separation. SE, sterol ester; TAG, triacylglycerol; Int. Std., internal standard (cholesteryl formate). Right: Quantification of sterol esters and triacylglycerol in μg/g wet weight. Compared by one-way ANOVA. ns, not significant; *, $p < 0.05$; ***, $p < 0.001$. n=3.

(E) Control and Δ*pex31* cells expressing Mig1-GFP were cultured to logarithmic growth phase on medium containing 2% glucose and subjected to SDS-PAGE and subsequent western blotting.

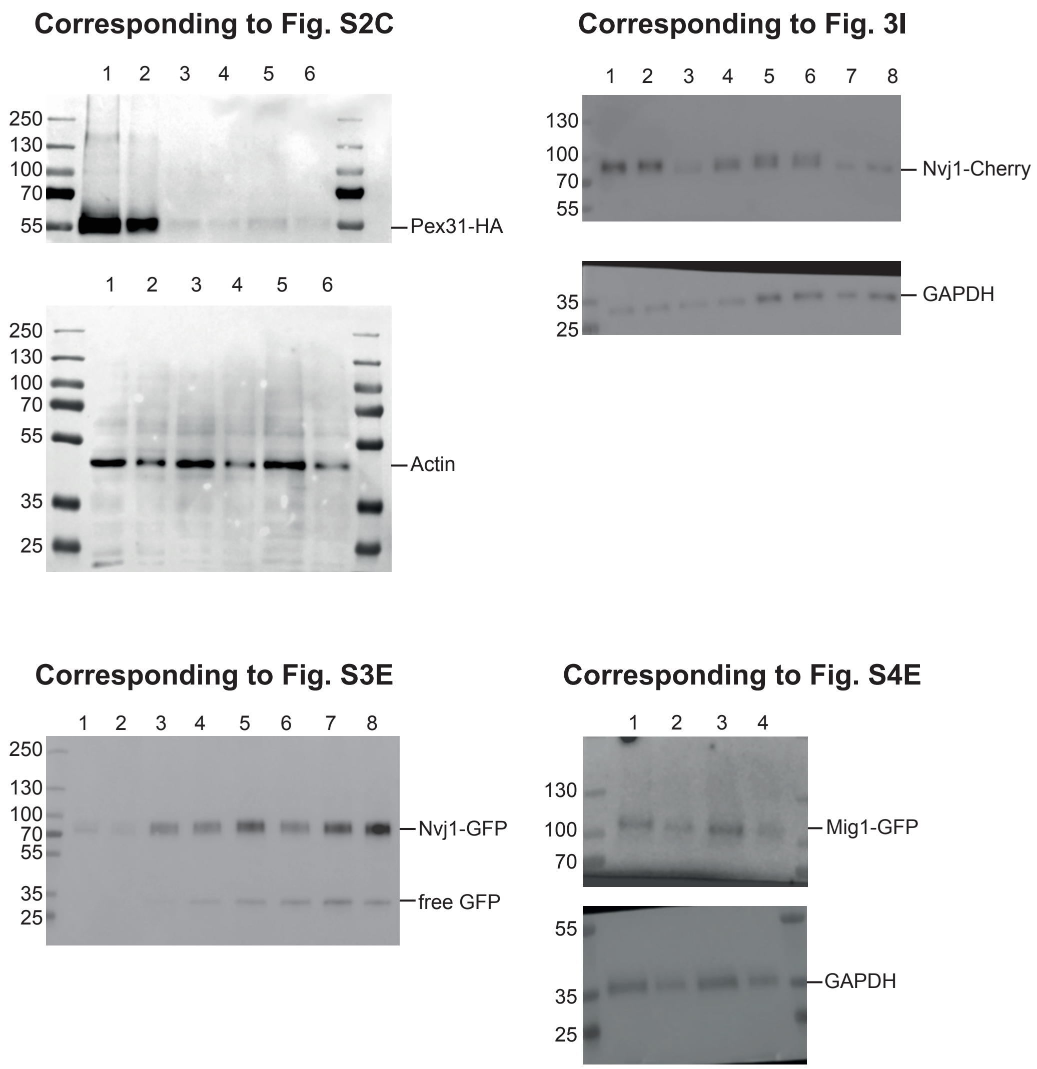

Fig. S5. Blot Transparency.

Top left: Corresponding to Fig. S2C. Marker in kDa on the left.

Top right: Corresponding to Fig. 3I. Marker in kDa on the left.

Bottom left: Corresponding to Fig. S3E. Marker in kDa on the left.

Bottom right: Corresponding to Fig. S4E. Marker in kDa on the left.

Table S1. Related to Fig. 1. List of genes analyzed in microscopy-based screen for NVJ resident proteins.

Available for download at
https://journals.biologists.com/jcs/article-lookup/doi/10.1242/jcs.264171#supplementary-data

Journal of Cell Science • Supplementary information

Table S2. List of yeast strains used in this study.

Name	Genotype	Source	Identifier
WT	his3Δ1 leu2Δ0 lys2+/lys+ met15Δ0 ura3Δ0 can1Δ::STE2pr-sp HIS5 lyp1Δ::STE3pr-LEU2	Breslow et al., 2008	yMB3
Nvj1-Cherry GFP-Shr5	his3Δ1 leu2Δ0 lys2+/lys+ met15Δ0 ura3Δ0 can1Δ::STE2pr-sp HIS5 lyp1Δ::STE3pr-LEU2 Nvj1-Cherry::NAT pNOP1-GFP-Shr5	This study	yMB1276
Nvj1-Cherry GFP-Nsg1	his3Δ1 leu2Δ0 lys2+/lys+ met15Δ0 ura3Δ0 can1Δ::STE2pr-sp HIS5 lyp1Δ::STE3pr-LEU2 Nvj1-Cherry::NAT pNOP1-GFP-Nsg1	This study	yMB1275
Nvj1-Cherry GFP-Nsg2	his3Δ1 leu2Δ0 lys2+/lys+ met15Δ0 ura3Δ0 can1Δ::STE2pr-sp HIS5 lyp1Δ::STE3pr-LEU2 Nvj1-Cherry::NAT pNOP1-GFP-Nsg2	This study	yMB1273
Nvj1-Cherry GFP-Tcb1	his3Δ1 leu2Δ0 lys2+/lys+ met15Δ0 ura3Δ0 can1Δ::STE2pr-sp HIS5 lyp1Δ::STE3pr-LEU2 Nvj1-Cherry::NAT pNOP1-GFP-Tcb1	This study	yMB1274
Nvj1-Cherry Pex31-mNG	his3Δ1 leu2Δ0 lys2+/lys+ met15Δ0 ura3Δ0 can1Δ::STE2pr-sp HIS5 lyp1Δ::STE3pr-LEU2 Nvj1-mCherry::NAT Pex31-mNG::HYG	This study	yMB1090
Δ*pex31*	his3Δ1 leu2Δ0 lys2+/lys+ met15Δ0 ura3Δ0 can1Δ::STE2pr-sp HIS5 lyp1Δ::STE3pr-LEU2 Δpex31::G418	This study	yMB273
GFP-NVJ2	his3Δ1 leu2Δ0 lys2+/lys+ met15Δ0 ura3Δ0 can1Δ::STE2pr-sp HIS5 lyp1Δ::STE3pr-LEU2 pNop1-GFP-Nvj2::URA	This study	yMB1001
GFP-NVJ2 Δ*pex31*	his3Δ1 leu2Δ0 lys2+/lys+ met15Δ0 ura3Δ0 can1Δ::STE2pr-sp HIS5 lyp1Δ::STE3pr-LEU2 Δpex31::G418 pNop1-GFP-Nvj2::URA	This study	yMB1002
Nvj1-Cherry Nsg1-GFP	his3Δ1 leu2Δ0 lys2+/lys+ met15Δ0 ura3Δ0 can1Δ::STE2pr-sp HIS5 lyp1Δ::STE3pr-LEU2 Nvj1-mCherry::Nat Nsg1-GFP::His	This study	yMB1011
Nvj1-Cherry Δ*pex31* Nsg1-GFP	his3Δ1 leu2Δ0 lys2+/lys+ met15Δ0 ura3Δ0 can1Δ::STE2pr-sp HIS5 lyp1Δ::STE3pr-LEU2 ΔPex31::G418 Nvj1-mCherry::Nat Nsg1-GFP::His	This study	yMB1013
Nvj1-Cherry Hmg2-GFP	his3Δ1 leu2Δ0 lys2+/lys+ met15Δ0 ura3Δ0 can1Δ::STE2pr-sp HIS5 lyp1Δ::STE3pr-LEU2 Nvj1-mCherry::Nat Hmg2-GFP::His	This study	yMB1010
Nvj1-Cherry Hmg2-GFP Δpex31	his3Δ1 leu2Δ0 lys2+/lys+ met15Δ0 ura3Δ0 can1Δ::STE2pr-sp HIS5 lyp1Δ::STE3pr-LEU2 Δpex31::G418 Nvj1-Cherry::NAT Hmg2-GFP::HIS3MX6	This study	yMB1020
Pex29-mNG Nvj1-Cherry	his3Δ1 leu2Δ0 lys2+/lys+ met15Δ0 ura3Δ0 can1Δ::STE2pr-sp HIS5 lyp1Δ::STE3pr-LEU2 Pex29-mNG::HYG Nvj1-mCherry::NAT	This study	yMB1117
Pex29-mNG Nvj1-mCherry Δ*pex31*	his3Δ1 leu2Δ0 lys2+/lys+ met15Δ0 ura3Δ0 can1Δ::STE2pr-sp HIS5 lyp1Δ::STE3pr-LEU2 Pex29-mNG::HYG Nvj1-mCherry::NAT Δpex31::G418	This study	yMB1118
Nvj1-Cherry Pex30-mNG Δpex31	his3Δ1 leu2Δ0 lys2+/lys+ met15Δ0 ura3Δ0 can1Δ::STE2pr-sp HIS5 lyp1Δ::STE3pr-LEU2	This study	yMB1188

Journal of Cell Science • Supplementary information

	Pex30-mNG::HYG Nvj1-mCherry::NAT Δpex31::G418		
Nvj1-Cherry Pex30-mNG	his3Δ1 leu2Δ0 lys2+/lys+ met15Δ0 ura3Δ0 can1Δ::STE2pr-sp HIS5 lyp1Δ::STE3pr-LEU2 Pex30-mNG::HYG Nvj1-mCherry::NAT	This study	yMB1159
Nvj1-Cherry	his3Δ1 leu2Δ0 lys2+/lys+ met15Δ0 ura3Δ0 can1Δ::STE2pr-sp HIS5 lyp1Δ::STE3pr-LEU2 Nvj1-Cherry::NAT	This study	yMB908
Nvj1-Cherry *Δpex31*	his3Δ1 leu2Δ0 lys2+/lys+ met15Δ0 ura3Δ0 can1Δ::STE2pr-sp HIS5 lyp1Δ::STE3pr-LEU2 Δpex31::G418 Nvj1-Cherry::NAT	This study	yMB900
Nvj1-Cherry *Δsnd3*	his3Δ1 leu2Δ0 lys2+/lys+ met15Δ0 ura3Δ0 can1Δ::STE2pr-sp HIS5 lyp1Δ::STE3pr-LEU2 Δsnd3::HYG Nvj1-mCherry::NAT	This study	yMB998
Nvj1-cherry *Δsnd3* *Δpex31*	his3Δ1 leu2Δ0 lys2+/lys+ met15Δ0 ura3Δ0 can1Δ::STE2pr-sp HIS5 lyp1Δ::STE3pr-LEU2 Nvj1-mCherry::G418 Δsnd3::NAT Δpex31::G418	This study	yMB1115
Snd3-GFP	his3Δ1 leu2Δ0 lys2+/lys+ met15Δ0 ura3Δ0 can1Δ::STE2pr-sp HIS5 lyp1Δ::STE3pr-LEU2Snd3-GFP::NAT	This study	yMB1119
Snd3-GFP *Δpex31*	his3Δ1 leu2Δ0 lys2+/lys+ met15Δ0 ura3Δ0 can1Δ::STE2pr-sp HIS5 lyp1Δ::STE3pr-LEU2 Snd3-GFP::NAT Δpex31::G418	This study	yMB1120
Pex30-mNG *Δsnd3*	his3Δ1 leu2Δ0 lys2+/lys+ met15Δ0 ura3Δ0 can1Δ::STE2pr-sp HIS5 lyp1Δ::STE3pr-LEU2 Pex30-mNG::NAT Δsnd3::HYG	This study	yMB1133
Pex30-mNGn *Δsnd3*Δpex31	his3Δ1 leu2Δ0 lys2+/lys+ met15Δ0 ura3Δ0 can1Δ::STE2pr-sp HIS5 lyp1Δ::STE3pr-LEU2 Pex30-mNG::NAT Δpex31::G418 Δsnd3::HYG	This study	yMB1134
Pex31-HA	his3Δ1 leu2Δ0 lys2+/lys+ met15Δ0 ura3Δ0 can1Δ::STE2pr-sp HIS5 lyp1Δ::STE3pr-LEU2 Pex31-HA::HIS	This study	yMB302
Zrc1-Cherry	his3Δ1 leu2Δ0 lys2+/lys+ met15Δ0 ura3Δ0 can1Δ::STE2pr-sp HIS5 lyp1Δ::STE3pr-LEU2Snd3-GFP::NAT Zrc1-Cherry::NAT	This study	yMB368
Vph1-mkate2	his3Δ1 leu2Δ0 lys2+/lys+ met15Δ0 ura3Δ0 can1Δ::STE2pr-sp HIS5 lyp1Δ::STE3pr-LEU2 Vph1-mKate2::NAT	This study	yMB813
Vph1-mKate2 *Δpex31*	his3Δ1 leu2Δ0 lys2+/lys+ met15Δ0 ura3Δ0 can1Δ::STE2pr-sp HIS5 lyp1Δ::STE3pr-LEU2 Vph1-mKate2::NAT Δpex31::G418	This study	yMB1114
OE-Pex31	his3Δ1 leu2Δ0 lys2+/lys+ met15Δ0 ura3Δ0 can1Δ::STE2pr-sp HIS5 lyp1Δ::STE3pr-LEU2 pTEF2-Pex31::URA	This study	yMB1471
OE-Pex31 Nvj1-Cherry	his3Δ1 leu2Δ0 lys2+/lys+ met15Δ0 ura3Δ0 can1Δ::STE2pr-sp HIS5 lyp1Δ::STE3pr-LEU2 pTEF2-Pex31::URA Nvj1-Cherry::Nat	This study	yMB1387
pTEF2-Pex31 Pex30 mNG Nvj1-Cherry	his3Δ1 leu2Δ0 lys2+/lys+ met15Δ0 ura3Δ0 can1Δ::STE2pr-sp HIS5 lyp1Δ::STE3pr-LEU2 pTEF2-Pex31::URA Nvj1-Cherry::Nat Pex30mNG::HYG	This study	yMB1218

Journal of Cell Science • Supplementary information

Nvj1-GFP	his3Δ1 leu2Δ0 lys2+/lys+ met15Δ0 ura3Δ0 can1Δ::STE2pr-sp HIS5 lyp1Δ::STE3pr-LEU2 Nvj1-GFP::NAT	This study	yMB921
Nvj1-GFP Δpex31	his3Δ1 leu2Δ0 lys2+/lys+ met15Δ0 ura3Δ0 can1Δ::STE2pr-sp HIS5 lyp1Δ::STE3pr-LEU2 Nvj1-GFP::NAT Δpex31::G418	This study	yMB1087
pNOP1-GFP-Gtt3	his3Δ1 leu2Δ0 lys2+/lys+ met15Δ0 ura3Δ0 can1Δ::STE2pr-sp HIS5 lyp1Δ::STE3pr-LEU2 pNOP1-GFP-Gtt3::URA	This study	yMB1474
Mig1-GFP	his3Δ1 leu2Δ0 lys2+/lys+ met15Δ0 ura3Δ0 can1Δ::STE2pr-sp HIS5 lyp1Δ::STE3pr-LEU2 Mig1-GFP::NAT	This study	yMB1482
Mig1-GFP Δpex31	his3Δ1 leu2Δ0 lys2+/lys+ met15Δ0 ura3Δ0 can1Δ::STE2pr-sp HIS5 lyp1Δ::STE3pr-LEU2 Mig1-GFP::NAT Δpex31::G418	This study	yMB1483
Δhmg2	his3Δ1 leu2Δ0 lys2+/lys+ met15Δ0 ura3Δ0 can1Δ::STE2pr-sp HIS5 lyp1Δ::STE3pr-LEU2 Δhmg2::NAT	This study	yMB1456
Δhmg2 Δpex31	his3Δ1 leu2Δ0 lys2+/lys+ met15Δ0 ura3Δ0 can1Δ::STE2pr-sp HIS5 lyp1Δ::STE3pr-LEU2 Δhmg2::NAT Δpex31::G418	This study	yMB1457

Journal of Cell Science • Supplementary information

Table S3. List of plasmids used in this study.

Name	Utilization	Source	Identifier
pFA6a-KanMX6	Yeast genomic manipulation (deletion)	Longtine et al., 1998	pMB5
pFA6a-NatMX6	Yeast genomic manipulation (deletion)	Goldstein and McCusker, 1999	pMB6
pFA6-Hygro	Yeast genomic manipulation (deletion)	Goldstein and McCusker, 1999	pMB10
pFA6a-HIS3	Yeast genomic manipulation (deletion)	Longtine et al., 1998	pMB49
pFA6a-NAT-Cherry	Yeast genomic manipulation (N-terminal tagging with Cherry)	Longtine et al., 1998	pMB9
pFA6a-NAT-eGFP	Yeast genomic manipulation (N-terminal tagging with eGFP)	Longtine et al., 1998	pMB12
pYM2 3HA-HIS3MX6	Yeast genomic manipulation (C-terminal tagging with 3xHA)	Janke et al., 2004	pMB230
pYM25-5xGA-mNeonGreen-HygR	Yeast genomic manipulation (C-terminal tagging with mNG)	Haase et al., 2023	pMB451
pYM42-5xGA-mNeonGreen-NatR	Yeast genomic manipulation (C-terminal tagging with mNG)	Haase et al., 2023	pMB91
pRS316-Pex31-FL-mNG	Pex31-mNG expression plasmid	This study	pMB474
pRS316-Pex31-ΔDysF-mNG	Pex31-ΔDysF-mNG expression plasmid	This study	pMB475
pRS316-Pex31-ΔRTN-mNG	Pex31-ΔRTN-mNG expression plasmid	This study	pMB476
pRS316-FFAT-Pex31-ΔRTN-mNG	FFAT-Pex31-ΔRTN-mNG expression plasmid	This study	pMB477
pRS316-Pex31-ΔRTN- Ubc6-mNG	Pex31-ΔRTN- Ubc6-mNG expression plasmid	This study	pMB478

Journal of Cell Science • Supplementary information

Table S4. List of primers used in this study.

Primer name	Sequence	Identifier
pMS80 chk F	ggcatggacgagctgtacaag	prMB22
pFA6 F1 rev com	TTAATTAACCCGGGGATCCG	prMB23
PEX31 5'UTR CHK F	TAATGTGGAGTGGGTAATCG	prMB162
PEX31 KO pFA6 F	CTGGTTGTCAAGCCTTGGTTTCCCTTTATTTGATA GTATGcggatccccgggttaattaa	prMB163
PEX31 KO pFA6 R	AGTGTGAACGTTGTTGTCCATATGGGGCATGCAC TCATTAgaattcgagctcgtttaaac	prMB164
PEX31 WT CHK F	GAATGGCACGATAAAGATTG	prMB165
PEX31 WT CHK R	TACTGTCCGAGGAAGGTATG	prMB166
Nvj1-Ctag-chk_F	TAAGGACATGAACGTTTTGG	prMB829
Nvj1-Ctag-pFA6_F	AGTGAACACTGAACAAGCATACTCTCAACCATTTA GATACcggatccccgggttaattaa	prMB830
Nvj1-Ctag-pFA6_R	GTGACGATGATAACCGAGATGACGGAAATATAGT ACATTAgaattcgagctcgtttaaac	prMB831
PEX31 C-tag CHK F	GAATGGCACGATAAAGATTG	prMB186
PEX31 C-tag pYM F	ATTAATACAAATATCTGATGTTTCAATGTCTCCTTC TCTAcgtacgctgcaggtcgac	prMB187
PEX31 C-tag pYM R	AGTGTGAACGTTGTTGTCCATATGGGGCATGCAC TCATTAatcgatgaattcgagctcg	prMB188
NOP1-NVJ2-gDNA-F	CTTTCATTTTTGCACCTAATTGGTATGGCACATTTT TCAGTTTTCTCCCACTTAAGTTTTC	prMB1491
NOP1-NVJ2-gDNA-R	TCTGCTGTATCATGATCTAGCTCCTTCTTCGATGC CGATTTAAGGTTACTGTACAATAAA	prMB1492
NVJ2 N-tag CHK R	GTTCTTTGCAAGATTTGCTC	prMB1493
HMG2_Ctag_CHK_F	ATTTGGTCACTGCACTTTTC	prMB1496
HMG2_Ctag_pFA6_F	AAGTAACAAAGGGCCCCCCTGTAAAACCTCAGCA TTATTAcggatccccgggttaattaa	prMB1497
HMG2_Ctag_pFA6_R	ACAAAGATATAAAGTATCACCATGTAAACTACAAG AGTTAgaattcgagctcgtttaaac	prMB1498
NSG1_Ctag_CHK_F	ACCATTCATTGACTCTTTCG	prMB1499
NSG1_Ctag_pFA6_F	GTTTTTGATGTTCAGCAAGTTGGGCAGATATTTAT TCAATcggatccccgggttaattaa	prMB1500
NSG1_Ctag_pFA6_R	CATCGATACTAATCATTGAACGCCCCTATGGGAAC ACTTAgaattcgagctcgtttaaac	prMB1501
PEX29 C-tag CHK F	GGATCCAAAAGAATGGGTAG	prMB1775
PEX29 C-tag pYM F	GTCAATCGAAGAGCTAACAGACACTCTCAATTCAA CTATAcgtacgctgcaggtcgac	prMB1776
PEX29 C-tag pYM R	TGTATCATCAGTGAACATATAGTATAACAAATCAA GTTTAatcgatgaattcgagctcg	prMB1777
PEX30 C-tag CHK F	GGTAAGAACCGCAGAATTG	prMB182
PEX30 C-tag pYM F	ATCAAATCCAACCATTGGTCGCGATAGCAAGAAG GCCGTAcgtacgctgcaggtcgac	prMB183
PEX30 C-tag pYM R	AGATTATATTATGTAAAGGTAAAAACGGGAGCGAG CATCAatcgatgaattcgagctcg	prMB184
SND3 C-tag CHK F	TCTTCCATCGGTATCTCTTG	prMB373

Journal of Cell Science • Supplementary information

SND3 C-tag pFA6 F	AGAAGCTGAAAGAGCCGGTAACGCTGGTGTTAAG GCTGAAcggatccccgggttaattaa	prMB374
SND3 C-tag pFA6 R	AAAACTAGGAAAAAAAAATACTTCGCTTTTGATCG AATCAgaattcgagctcgtttaaac	prMB375
VPH1-C-tag-pFA6_F	GGAAGTCGCTGTTGCTAGTGCAAGCTCTTCCGCT TCAAGCcggatccccgggttaattaa	prMB1002
VPH1-C-tag-pFA6_R	ACTTAAATGTTTCGCTTTTTTTAAAAGTCCTCAAAA TTTAgaattcgagctcgtttaaac	prMB1003
pTEF2-PEx31-F	CTGGTTGTCAAGCCTTGGTTTCCCTTTATTTGATA GTATGCGGATCCCCGGGTTAATTAA	prMB347
pTEF2-Pex31 Rev	TACTTGAGGTTGGCTCTAGATTTTCATTATTTATTT CGCTCATGGTACTAGTGTTTAGTTAAT	prMB2162
MIG1 C' tag CHK F	GCAAGACTATTTGCAAGAGC	prMB2367
MIG1 C' tag pFA6 F	ACCCATAAGAAGTTTACCGTTGCCCTTCCCACACA TGGACcggatccccgggttaattaa	prMB2368
MIG1 C' tag pFA6 R	TTTGATTTATCTGCACCGCCAAAAACTTGTCAGCG TATCAgaattcgagctcgtttaaac	prMB2369
Pex31_bckbn_Fwd	GCATGGACGAGCTGTACAAGtaatgagtgcatgccccata	prMB2370
Pex31_bckbn_Rev	CCAGCACCAGCACCTGCTCCtagagaaggagacattgaa aca	prMB2371
mNG_Fwd	tttcaatgtctccttctctaGGAGCAGGTGCTGGTGCTGG	prMB2372
mNG_Rev	tatggggcatgcactcattaCTTGTACAGCTCGTCCATGCC	prMB2373
PEx31_bckb_dDys_Rev	CCAGCACCAGCACCTGCTCCtccaccaaggtctaatcctg	prMB2374
dDys_mNG_Fwd	caggattagaccttggtggaGGAGCAGGTGCTGGTGCTGG	prMB2375
Pex31_bckbn_dRTN_rev_1	aagcgcctattattatctggcatactatcaaataaagggaaacca	prMB2376
Pex31_bckbn_dRTN_fwd_1	tccctttatttgatagtatgccagataataataggcgcttg	prMB2377
Pex31_FFAT_bckbn_Rev	TTTCCATCTTGCTTGTGCAAcatactatcaaataaagggaa acca	prMB2378
FFAT_Fwd	tccctttatttgatagtatgTTGCACAAGCAAGATGGAAA	prMB2379
FFAT_Rev	aagcgcctattattatctggTTTCACCACAATAGACTGCT	prMB2380
Pex31_FFAT_bckbn_Fwd	AGCAGTCTATTGTGGTGAAAccagataataataggcgcttg	prMB2381
Pex31_Bckbn_Ubc6_Fwd	GCCCTACTTCTCAAACCAGAtagagaaggagacattgaaa ca	prMB2382
Ubc6_Fwd	tttcaatgtctccttctctaTCTGGTTTGAGAAGTAGGGC	prMB2383
Ubc6_Rev	CCAGCACCAGCACCTGCTCCTTTCATAAAAAGGC CAACCAA	prMB2384
mNG_Ubc6_Fwd	TGGTTGGCCTTTTTATGAAAGGAGCAGGTGCTGG TGCTGG	prMB2385
HMG2 5'UTR CHK F	ACCATTGATCGTTAGAGACG	prMB1093
HMG2 KO pFA6 F	CAAATTAGTTCAACAAGGTTCCCACATACAACCTC AAATGcggatccccgggttaattaa	prMB1094
HMG2 KO pFA6 R	ACAAAGATATAAAGTATCACCATGTAAACTACAAG AGTTAgaattcgagctcgtttaaac	prMB1095
HMG2 WT CHK F	TGAGATTTCGGACAACTACC	prMB1096
HMG2 WT CHK R	AGTGCAGTGACCAAATTAGC	prMB1097

Journal of Cell Science • Supplementary information

Table S5. List of antibodies used in this study.

Antibodies	Source	Identifier	Dilution
Cherry	ThermoFisher	PA5-34974	1:1000
GAPDH	Abcam	Cat#125247; RRID: AB_11129118	1:2000
HA	Proteintech	Cat# 51064-2-AP	1:1000
Actin	MP Biomedicals	08691001	1:1000
GFP	Sigma	11814460001	1:500
anti-mouse IgG	Dianova	Cat#115-035-003	1:10000
anti-rabbit IgG	Dianova	Cat#111-035-003	1:10000

References

Breslow, D. K., Cameron, D. M., Collins, S. R., Schuldiner, M., Stewart-Ornstein, J., Newman, H. W., Braun, S., Madhani, H. D., Krogan, N. J. and Weissman, J. S. (2008). A comprehensive strategy enabling high-resolution functional analysis of the yeast genome. *Nat Methods*. **5**, 711–718. doi:10.1038/nmeth.1234.

Goldstein, A. L. and McCusker, J. H. (1999). Three new dominant drug resistance cassettes for gene disruption inSaccharomyces cerevisiae. *Yeast*. **15**, 1541–1553. doi:10.1002/(SICI)1097-0061(199910)15:14<1541::AID-YEA476>3.0.CO;2-K.

Haase, D., Rasch, C., Keller, U., Tsytsyura, Y., Glyvuk, N., Elting, A., Wittmar, J., Janning, A., Kahms, M. and Wedlich, N. et al. (2023). Tetraspanner-based nanodomains modulate BAR domain-induced membrane curvature. *EMBO reports*. **24**, e57232. doi:10.15252/embr.202357232.

Janke, C., Magiera, M. M., Rathfelder, N., Taxis, C., Reber, S., Maekawa, H., Moreno-Borchart, A., Doenges, G., Schwob, E. and Schiebel, E. et al. (2004). A versatile toolbox for PCR-based tagging of yeast genes: new fluorescent proteins, more markers and promoter substitution cassettes. *Yeast (Chichester, England)*. **21**, 947–962. doi:10.1002/yea.1142.

Longtine, McKenzie III, Demarini and Shah, Wach, Brachat, Philippsen and Pringle (1998). Additional modules for versatile and economical PCR-based gene deletion and modification in Saccharomyces cerevisiae. **1998**.

Journal of Cell Science • Supplementary information
